# Supplementary material for: Thiol Reductases in Deinococcus Bacteria and Roles in Stress Tolerance
Source: Antioxidants (Basel). 2022 Mar 16;11(3):561. doi: 10.3390/antiox11030561 (PMC8945050; doi:10.3390/antiox11030561)
Supplement: Supplementary file 1 [file antioxidants-11-00561-s001.zip › antioxidants-1619302-supplementary.pdf]

Supplementary Table S1 and Supplementary Figures S1 to S15 for:

Thiol reductases in *Deinococcus* bacteria and roles in stress tolerance

by:

Arjan de Groot, Laurence Blanchard, Nicolas Rouhier and Pascal Rey

Table S1. Locus tags of genes encoding thiol reductases and related proteins from seven *Deinococcus* species.

Figure S1. Multiple sequence alignment of *Deinococcus*, *E. coli* and *B. subtilis* NADPH thioredoxin reductases.

Figure S2. Multiple sequence alignment of *Deinococcus*, *E. coli* and *B. subtilis* thioredoxins.

Figure S3. Gene clusters with additional *trxR*, *trxA* and *ahpD* genes in *D. peraridilitoris*.

Figure S4. 3D models of Trx and Trx-like proteins of *D. radiodurans*.

Figure S5. Sequence alignments of Trx-like proteins from *Deinococcus.*

Figure S6. Multiple sequence alignment of *Deinococcus* FrnE reductases.

Figure S7. Putative operons encoding DsbA, DsbB and UvrA.

Figure S8. Multiple sequence alignments of *Deinococcus* Dsb oxidoreductases.

Figure S9. Gene clusters encoding CcdA and DsbE/CcmG homologs.

Figure S10. Multiple sequence alignment of *Deinococcus* Msr proteins.

Figure S11. Multiple sequence alignment of *Deinococcus* AhpE and AhpD proteins.

Figure S12. 3D models of BCPs and OsmC of *D. radiodurans.*

Figure S13. Multiple sequence alignment of *Deinococcus* OsmC, Ohr and YhfA proteins.

Figure S14. Multiple sequence alignment of *Deinococcus* bacillithiol reductases.

Figure S15. Multiple sequence alignment of *Deinococcus* bacilliredoxins.

Table S1. Locus tags of genes encoding thiol reductases and related proteins from seven *Deinococcus* species.

**Name / description**	***D. radiodurans***	***D. deserti***	***D. geothermalis***	***D. gobiensis***	***D. maricopensis***	***D. peraridilitoris***	***D. proteolyticus***
**Thioredoxin reductase, thioredoxins and thioredoxin-like proteins**							
TrxR	DR_1982	Deide_05800	Dgeo_1576<br>Dgeo_2772	DGo_CA2339	Deima_1454	Deipe_0175<br>Deipe_3902	Deipr_0873
TrxA (Trx1)	DR_0944	Deide_18600	Dgeo_1837	DGo_CA0861	Deima_2910	Deipe_3068<br>Deipe_3873<br>Deipe_3901	Deipr_0424
TrxC (Trx2) (zinc site)	DR_A0164	Deide_01140	Dgeo_2518				
Trx-like	DR_2085	Deide_06390	Dgeo_1508	DGo_CA2073	Deima_1013	Deipe_0695	Deipr_0576
Trx-like	DR_0057	Deide_13741	Dgeo_0729	DGo_CA0407	Deima_1186	Deipe_0565	Deipr_1451
Trx-like	DR_A0072		Dgeo_2583	DGo_PC0201<br>DGo_PC0211			Deipr_2424<br>Deipr_2732
Trx-like	DR_B0110		Dgeo_1776	DGo_PA0204			Deipr_2190
Trx-like	DR_0948	Deide_06780	Dgeo_1960	DGo_CA2541	Deima_2994		Deipr_1792
**Other thiol-based disulfide oxidoreductases, (predicted) cytoplasmic**							
FrnE	DR_0659	Deide_00690	Dgeo_2073	DGo_CA0380	Deima_0892	Deipe_2202	Deipr_1901
FrnE-like		Deide_3p01230					Deipr_1559
DSBA-like thioredoxin domain-containing protein	DR_2335	Deide_22890		DGo_CA0030	Deima_0620	Deipe_1019	
**Other thiol-based disulfide oxidoreductases, (predicted) periplasmic or cytoplasmic membrane**							
DsbA family protein; DsbA? DsbC? DsbG?	DR_2019<br>DR_0560	Deide_06420	Dgeo_0747	DGo_CA1399	Deima_1134		Deipr_0943
DsbA family protein; DsbA? DsbC? DsbG?	DR_0753	Deide_12740	Dgeo_0692	DGo_CA1008	Deima_1749		Deipr_0493 (& Deipr_2421?)
DsbB family	DR_0754	Deide_12730	Dgeo_0691	DGo_CA1007	Deima_1748		Deipr_0492
DsbD family; CcdA	DR_1300	Deide_08350	Dgeo_1241	DGo_CA1639	Deima_1155	Deipe_0794	Deipr_0982
TlpA-like family; DsbE/CcmG	DR_0345<br>DR_0189	Deide_08290	Dgeo_1248	DGo_CA2017<br>DGo_PC0193	Deima_1148 (& Deima_1627 ?)	Deipe_0801<br>Deipe_4366	Deipr_0892
DsbD family; CcdA		Deide_2p00430				Deipe_0661	
TlpA-like family; DsbE/CcmG		Deide_2p00420				Deipe_0660	
**Thioredoxin-dependent methionine sulfoxide reductases, cytoplasmic**							
MsrA	DR_1849	Deide_10980	Dgeo_0843	DGo_CA1541	Deima_1788	Deipe_3499	Deipr_1412
MsrB	DR_1378	Deide_04050	Dgeo_2072	DGo_CA0919	Deima_1441	Deipe_4299	Deipr_1900

**Potential Mo-dependent sulfoxide reductases, predicted cytoplasmic**							
Molybdopterin oxidoreductase family protein	DR_0397	Deide_18410	Dgeo_0402	DGo_CA0112	Deima_0538	Deipe_1423	Deipr_1886
Sulfite oxidase family, molybdopterin binding domain	DR_0716	Deide_17540	Dgeo_1719	DGo_CA1115	Deima_0813	Deipe_2833	Deipr_0695
**Mo-dependent methionine sulfoxide reductase system, periplasmic**							
MsrP	"DR_2536" (frameshift)	Deide_20380	Dgeo_0877	DGo_CA2733	Deima_3114	Deipe_2978	Deipr_1129
MsrQ	DR_2537	Deide_20370	Dgeo_0878	DGo_CA2734	Deima_3115	Deipe_2977	Deipr_1128
**Thioredoxin-dependent peroxidases**							
PRX_BCP	DR_0846	Deide_10900	DgeoAM_1323 (= WP_041221145.1)	DGo_CA1364	Deima_2368	Deipe_0259	Deipr_0703
PRX_BCP	DR_1209<br>DR_1208	Deide_09051	Dgeo_0990<br>Dgeo_2729	DGo_CA1403	Deima_1714<br>Deima_0169	Deipe_3580<br>Deipe_3178	Deipr_1557
PRX_BCP		Deide_23291 ? (partial?)		DGo_CA0314			
**Alkyl hydroperoxide reductases**							
AhpE (PRX_AhpE_like)	DR_2242	Deide_02430	Dgeo_0122	DGo_CA2657	Deima_0618	Deipe_1016	Deipr_0175
AhpD-like	DR_1765	Deide_13030<br>Deide_1p00700	Dgeo_1446	DGo_CA1027	Deima_0298	Deipe_3296<br>Deipe_4199<br>Deipe_3903<br>Deipe_3878<br>Deipe_3900	Deipr_2741
**OsmC/Ohr/YhfA family proteins**							
OsmC	DR_1538	Deide_16090	Dgeo_0526	DGo_CA1241	Deima_0667	Deipe_3743	
Ohr	DR_1857		Dgeo_0446	DGo_CA0901<br>DGo_CA1828	Deima_2331<br>Deima_0137	Deipe_0225	Deipr_0815<br>Deipr_0816
YhfA	DR_1177	Deide_10790<br>Deide_21170	Dgeo_1268	DGo_CA1763	Deima_2343	Deipe_0234<br>Deipe_0648	Deipr_0697
**Bacillithiol disulfide reductase, bacilliredoxin**							
Bdr	DR_2623	Deide_23360	Dgeo_2331	DGo_CA0078	Deima_0670	Deipe_2475	Deipr_1732
Brx (AbxC)	DR_1832	Deide_14700	Dgeo_1464	DGo_CA1021	Deima_1446	Deipe_3166	Deipr_0555

Figure S1

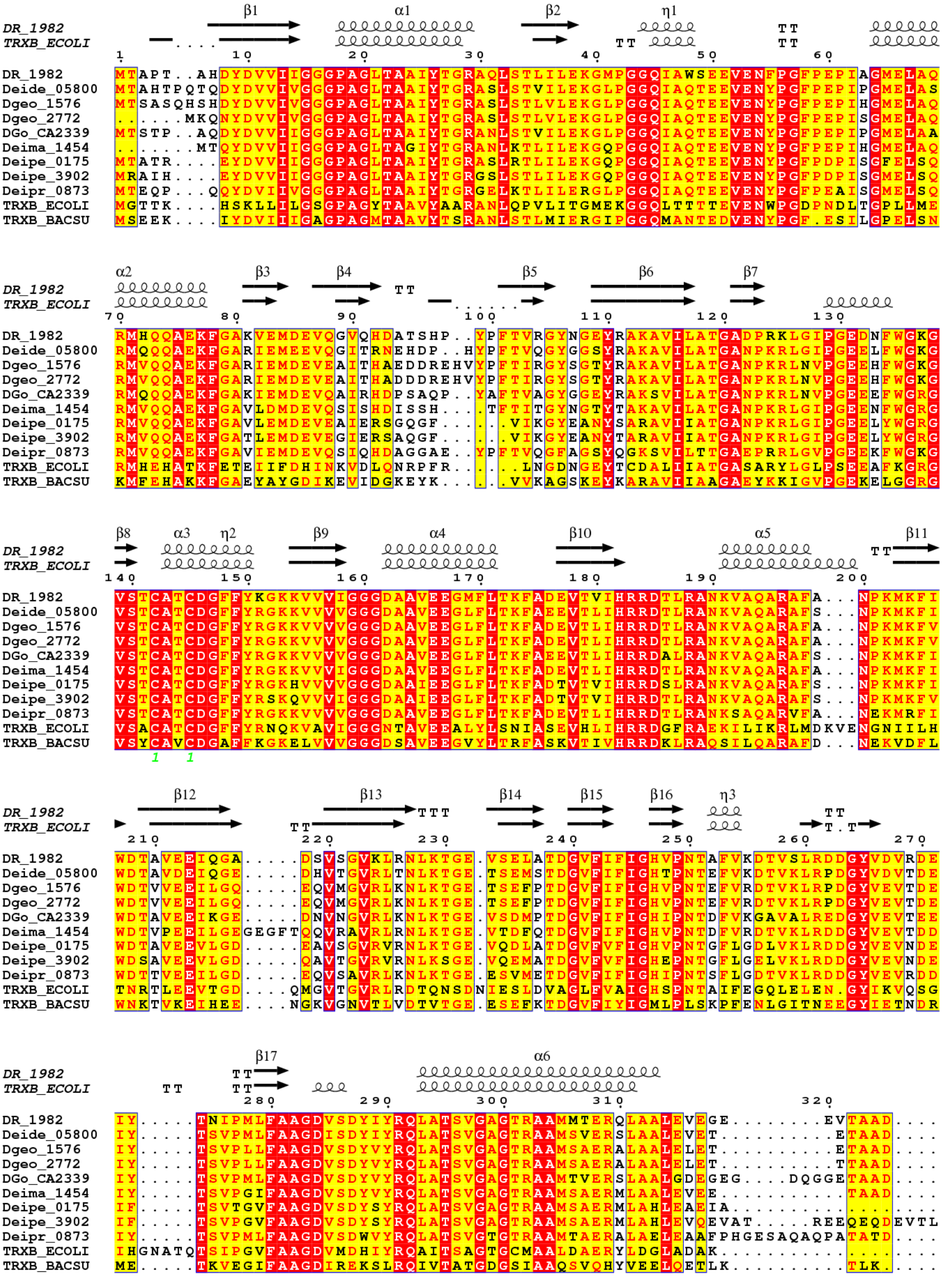

Figure S1. Multiple sequence alignment of *Deinococcus*, *E. coli* and *B. subtilis* NADPH thioredoxin reductases. Abbreviations of *Deinococcus* species names (*i.e.* the first part of the locus tags (gene numbers)) are as specified in Table 1. TRXB_ECOLI and TRXB_BACSU, thioredoxin reductases from *E. coli* and *B. subtilis*, respectively. The alignment was made with ClustalW at NPS@ [178] and ESPript [179].

Figure S2

(a)

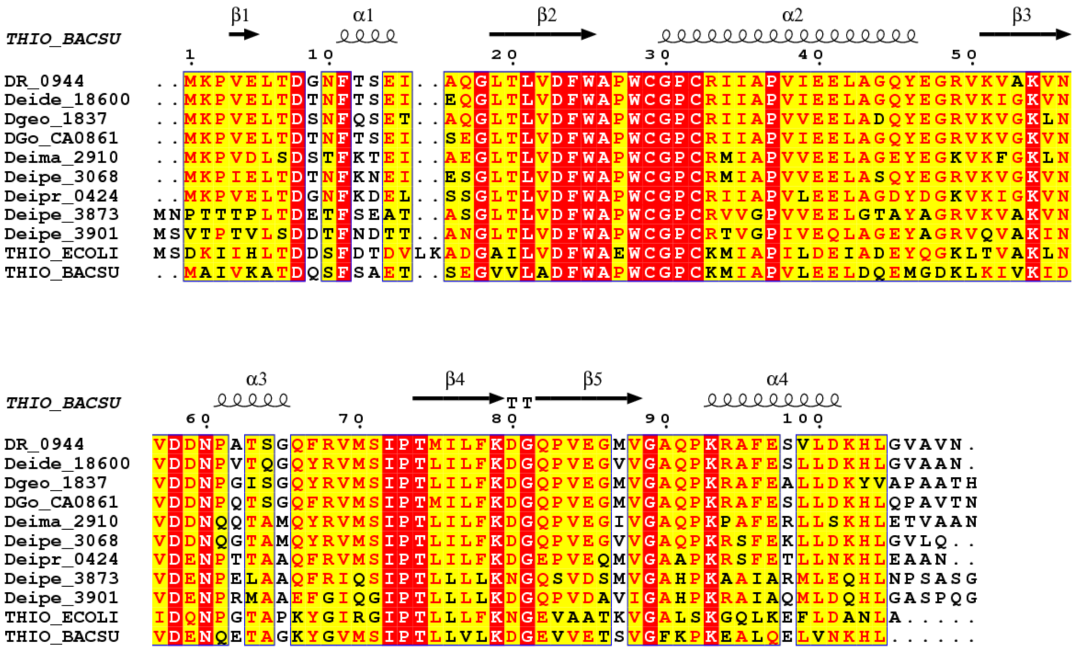

(b)

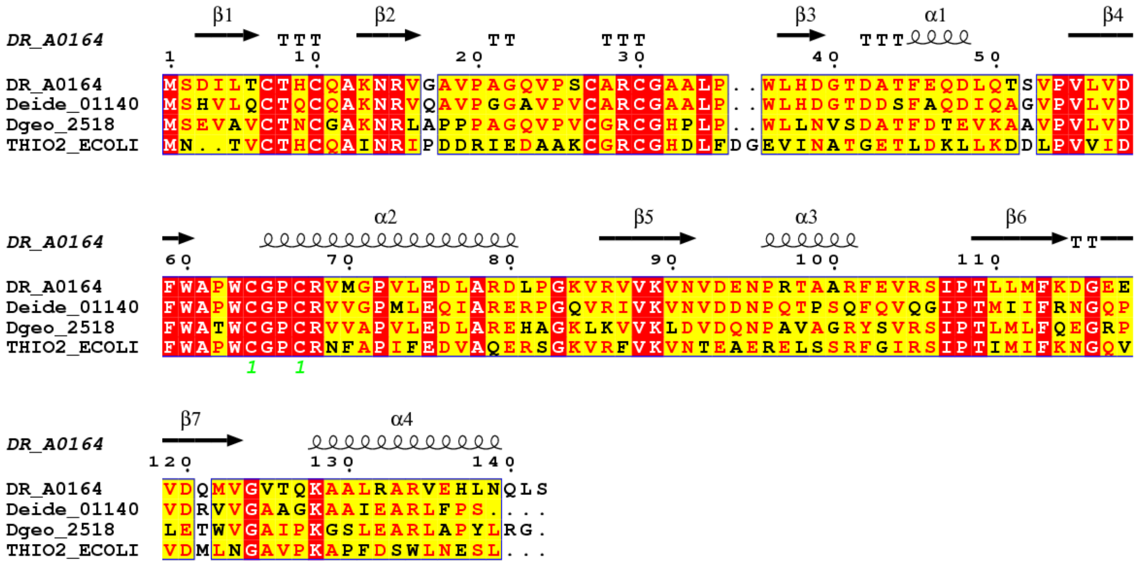

Figure S2. Multiple sequence alignment of *Deinococcus*, *E. coli* and *B. subtilis* thioredoxins. **(a)** Alignment of Trx1 sequences. **(b)** Alignment of Trx2 sequences. Abbreviations of *Deinococcus* species names are as specified in Table 1. THIO_ECOLI and THIO_BACSU, thioredoxins 1 from *E. coli* and *B. subtilis*, respectively. THIO2_ECOLI, thioredoxin 2 from *E. coli*. Alignment was made as in Figure S1.

Figure S3

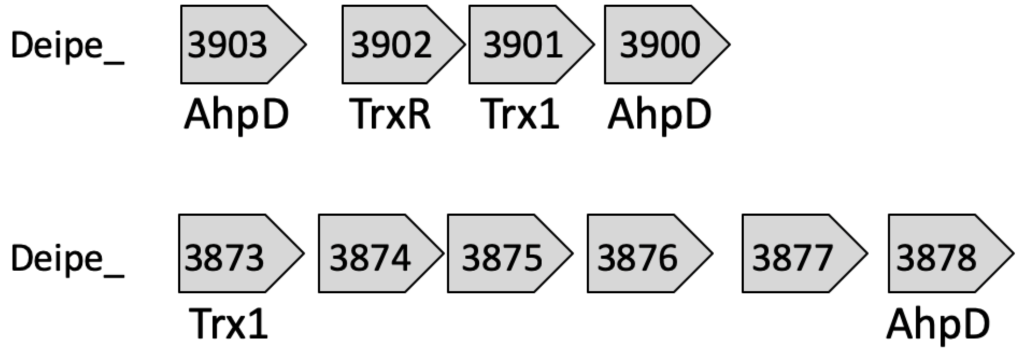

Figure S3. Gene clusters with additional *trxR*, *trxA* and *ahpD* genes in *D. perraridilitoris*. The two clusters shown are both located on plasmid pDEIPE01. Locus tags (Deipe_3903 etc.) are indicated. Gene sizes not drawn to scale.

Figure S4

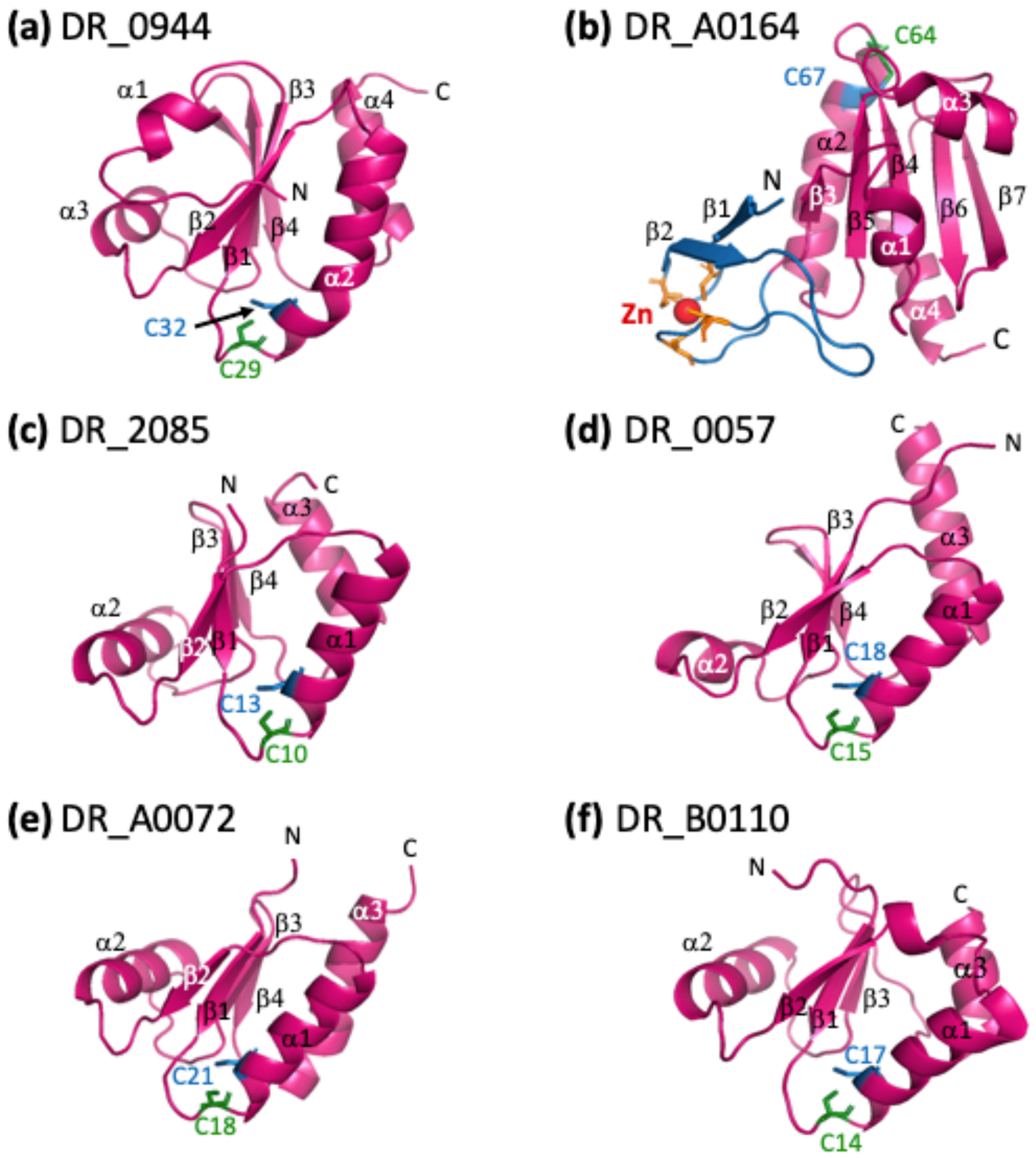

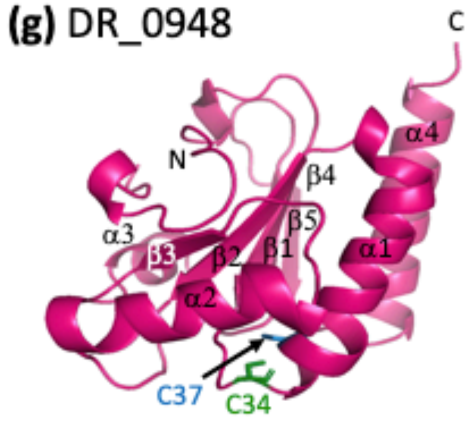

Figure S4. 3D models of Trx and Trx-like proteins of *D. radiodurans*. Models in panels **(a)** (Trx1) and **(c)** to **(g)** (Trx-like) were obtained with AlphaFold2_ Advanced [180]. **(b)** Crystal structure of Trx2 (PDB 7DL6) showing the extra N-terminal zinc binding domain in dark blue [60]. Catalytic and resolving cysteine residues are shown in green and blue, respectively. 3D structure or model images were generated using PyMOL (PyMOL Molecular Graphics System, Version 2.0 Schrödinger, LLC).

Figure S5 (panels a to e)

(a)

```
DR_0057     --------------MTPLP-TLTLYHRPGCHLCEQAQTHLDELGFAYQLHDISGDAALTA 45
Deide_13741 MTGRTGPDQQDPVQRPAVP-ALTLYSRLGCHLCEQAESHLKALAFTFQVVDVDTDPLLKA 59
Dgeo_0729   -----------------MLP-VLTLYTRAGCHLCEQAQANLQALEYRYEPLDVDRVPALQA 43
DGo_CA0407  ----------------MPEP-GLRLYSRVGCHLCEQAEERLDRYGFEYERLEVSGDPERER 44
Deima_1186  --------------MTAAP-TLTLYTRAGCHLCEDAEATLTRLGVPYTPVDVTGHADLEA 45
Deipe_0565  ----MAPA-ELKAGQPGTQ-GLVLYSRAGCHLCEDAERMLASLSVPFTRIEVSSDDDLER 54
Deipr_1451  MTD----S--AAAPLPSTPLIPVFYTRASCKLCQQAEALLRAWEVPFQRVDIAGDEDLIA 54
                                   :* * .*:**::*:  *      :   ::

DR_0057     RHGDHIPVLALG------DRVLLRGVLSRSRLSTLKLRLLREQEA--------- 84
Deide_13741 RYGQDVPVLASG------GRVLGKGAFSRSRLSQIKLLLLRETRTQPDSPG--- 104
Dgeo_0729   RYGDDVPVLALG------ERVLLKGVLSRGRLSALKLQLLREQAGQRKRV---- 87
DGo_CA0407  LYGHDVPVLTDR-----AGRVLLRGVFGPGRLGELRLRLRRELAP--GGAGDV- 90
Deima_1186  RYGWDVPVLARG------DQVLLKGVLSAARITA-KLRVHRLT----------- 81
Deipe_0565  RYGWDVPVLTRSPSAGHPPEVLLKGVFSRARILARLA-------GRP------- 94
Deipr_1451  RYGHHVPVLTLPLPGG--ERTLHRGPLTRSSLPALQLRLIRLRRELSSAPRQLH 106
             :* .:***:         ..* :* :  . :
```

(b)

```
DR_A0072   MPERSPAPDITVYTVPDCPDCEAVQALLRRRGLTPTVRNVRGDPAALAEMQRRADGVRIA 60
Dgeo_2583  -----MSMTVTVYTVPNCSSCEAVKRFLRSRGVPFTEKNIREDPAALAEMQARA-NVRIA 54
Deipr_2424 ----MKNLEVTLYTVPDCADCEAIKRLLKRENVPFTEKNVRGDPEALAEMQLRADGVRIA 56
DGo_PC0211 ------MPDITLYTVPQCADCEAIKRLLQHEGAPFTEKNVRGDPQALAEMQRRA-DVRIA 53
DGo_PC0201 ------MKTVTVYTVPGCASCEAIKRFLAARRVPYTEKNVREDPAALAEMQAKA-RVRIA 53
Deipr_2732 ------MPKVILYATPTCPDCHALRLWFNRKGIEFEERNLTIP-AVADEAKA-RYGVRVA 52
                    : :*:.* * .*.*::  :  .      :*:     .  * :     **:*

DR_A0072   PVTIIGEQVFYGPFDEQRPRLLAALERQGTQ- 91
Dgeo_2583  PVTVIGDQAFYGTFDDQRPLLEAALGENGI-- 84
Deipr_2424 PVTMIGQQAFYGRFDEQRPQILAALAEAQNHG 88
DGo_PC0211 PVTIIDEHIFYGPFSDQRPRILAALEKRA--- 82
DGo_PC0201 PVTVIGEEAFFGTFDDQRPFLEAALRENDA-- 83
Deipr_2732 PITVVGDQFFYGTFEQQRPELEPLFA------ 78
           *:*::.:. *:* *.:*** :   :
```

(c)

```
DR_B0110   ----MSDRPFLLLTQGACPGCERLKKMLAGPLRGQFDSHIEVIHRQSAQERFDALSAHFG 56
Dgeo_1776  ----------MLLTQANCPACVRLERMLSGPLRGAYWEQIEVVRREDDERQFLALAGEYG 50
DGo_PA0204 MTDPAPKAEVLMLTQDACPDCERLKLMLDKPLRGQFAGLIRPVHRQGQPDEFESVVALYG 60
Deipr_2190 MTEPAVQTRFVLLTQDNCPNCERLKLMLAKPLRGQFDDQIVTVHREQAASEFEALAAEYS 60
                     ::***  ** * **: **  **** :   *  ::*:    .* :: . :.

DR_B0110   VRSVPALIRVSDGTRAHDPGSLGAVRAFLQG--------- 87
Dgeo_1776  VRSTPALVERGTGRQITGAGSLHEVRLLLGV--------- 81
DGo_PA0204 VQKTPALIDTASGRVLLNTGGLGEVKAFLTAAQVAEPVGS 100
Deipr_2190 VQSTPALIDRERGEVLRNTGGLGEVKNFLTA--------- 91
           *:..***:    *    . *.*  *: :*
```

(d)

```
DR_0948     --------MRWPDPADFVHGSPLPPPTEWGRPGLLMTFNLECPGCVSRGIPFLKRLHAEY 52
Deide_06780 --------MEWPSQEDFVHGDPVAPPCGWDRPGLVMTFNLECSGCVSRGIPFLKRLHSEF 52
Dgeo_1960   --------MDWPAPEDFVHGDPLPPPDQWTRPGLVMVFNLECPGCVSRGLPFLRQLHTEF 52
DGo_CA2541  --------MLWPAPADFVHGSPLPPPTGWTRPGLVMTFNLECPGCVSRGVPFLKRLHGEF 52
Deima_2994  -------MTLWPSIDTFVHGTPVPSPEQVNRPMLVMFFNLECAGCVSRGIPFMKRLHAEF 53
Deipr_1792  MTPESSPPLPWPAAGDFVWRAADVD---PARPSLLMFFHLECAGCVSRGIPFMKRLHAEY 57
                      **    **            ** *:* *:*** ******:**:::** *:

DR_0948     GEQVHLLAVHTSFGHRQLTREEVEPTLVKFARDFAKLPFPVALDLDGSFAREWQTEGTPH 112
Deide_06780 GGRVQLLAVHTSWGHRQLPREDVEPTLLKFSRDFARLPFPVALDLDGSFARHWNTEGTPH 112
Dgeo_1960   GDRVHLLALHTSRGHRRLPREDVEPTLVRFAREYARLPFPVALDLEGDLARTWATEGTPH 112
DGo_CA2541  GDRVNLLAVHTSLGHRDLARGDVEPTLVKFARDFARLPFAVALDLDGSFARHWHTEGTPY 112
Deima_2994  GEAVQMLVIHTAHGHRQLPRQDVEPTLVRFAQSFAKLPFPVALDLDGEIAQAWRTEGTPH 113
Deipr_1792  GEQVNFIAVHTSRGHRQLPRADILPTLLHFAERFARLPFPVALDETGALAAAYATEGTPH 117
            *  *:::.:**: *** * * :: ***::*:. :*:*** ****  * :*  : *****:

DR_0948     WLAFAPGGELLRSVYGSQENAQTRLEYLLAEWAAASRP- 150
Deide_06780 WLAFAPGGELLRSVYGSQENAQTRLQYLLQEWAGQGDGP 151
Dgeo_1960   WLAFAAGGELLRSVYGSQENAQTRLQYLLEELVGPA--- 148
DGo_CA2541  WLAFAPGGELLRSVYGSQENAQTRLQYLLEEWTGQAEE- 150
Deima_2994  WLVFGAGGELLRSLYGSQENAQTRLEYLLAELATSS--- 149
Deipr_1792  WIAL-EGGEVVRSIYGSQENAQTRLEYWLAELTSAGDA- 154
            *:.:  ***::**:***********:* * * .  .
```

(e)

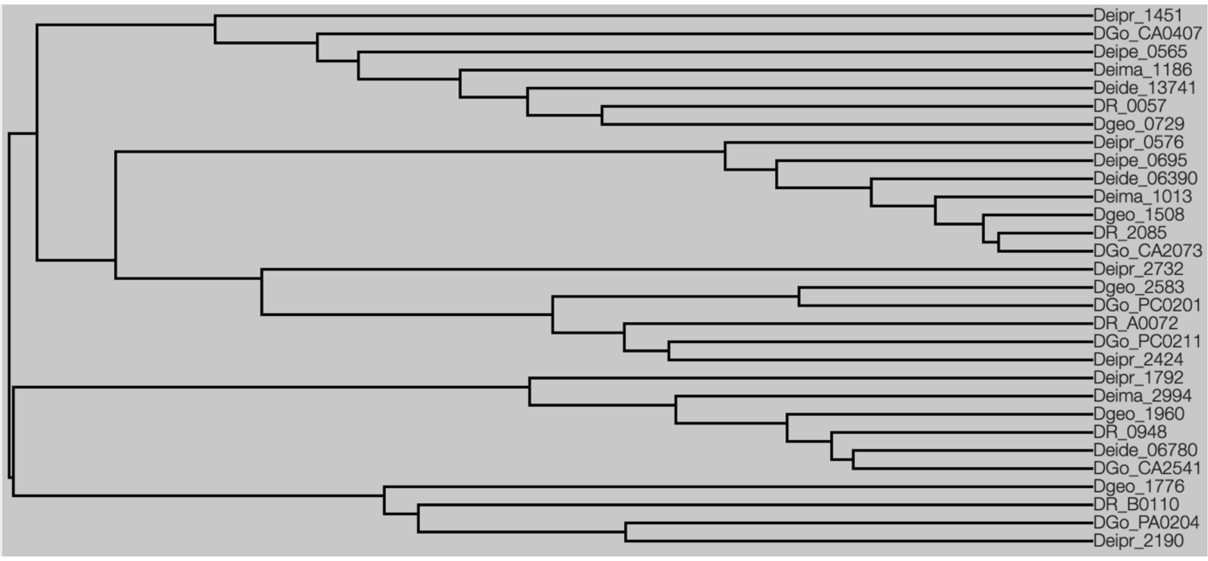

Figure S5. Sequence alignments of Trx-like proteins from *Deinococcus.* **(a)** Alignment of *Deinococcus* DR_0057-type sequences. **(b)** Alignment of *Deinococcus* DR_A0072-type sequences. **(c)** Alignment of *Deinococcus* DR_B0010-type sequences **(d)** Alignment of *Deinococcus* DR_0948-type sequences. **(e)** Tree showing sequence relationship between Trx-like proteins shown in Figure 2 and in panels (a)-(d). The guide tree is calculated based on the distance matrix that is generated from the pairwise scores. Trx active site sequences are highlighted in yellow, residues highlighted in cyan in Deipr sequences differ in the proximity of the active site. Abbreviations of *Deinococcus* species names are as specified in Table 1. Alignments and tree were obtained with UniProt ClustalO (https://www.uniprot.org/align/) [181].

Figure S6 (panels a to c)

(a)

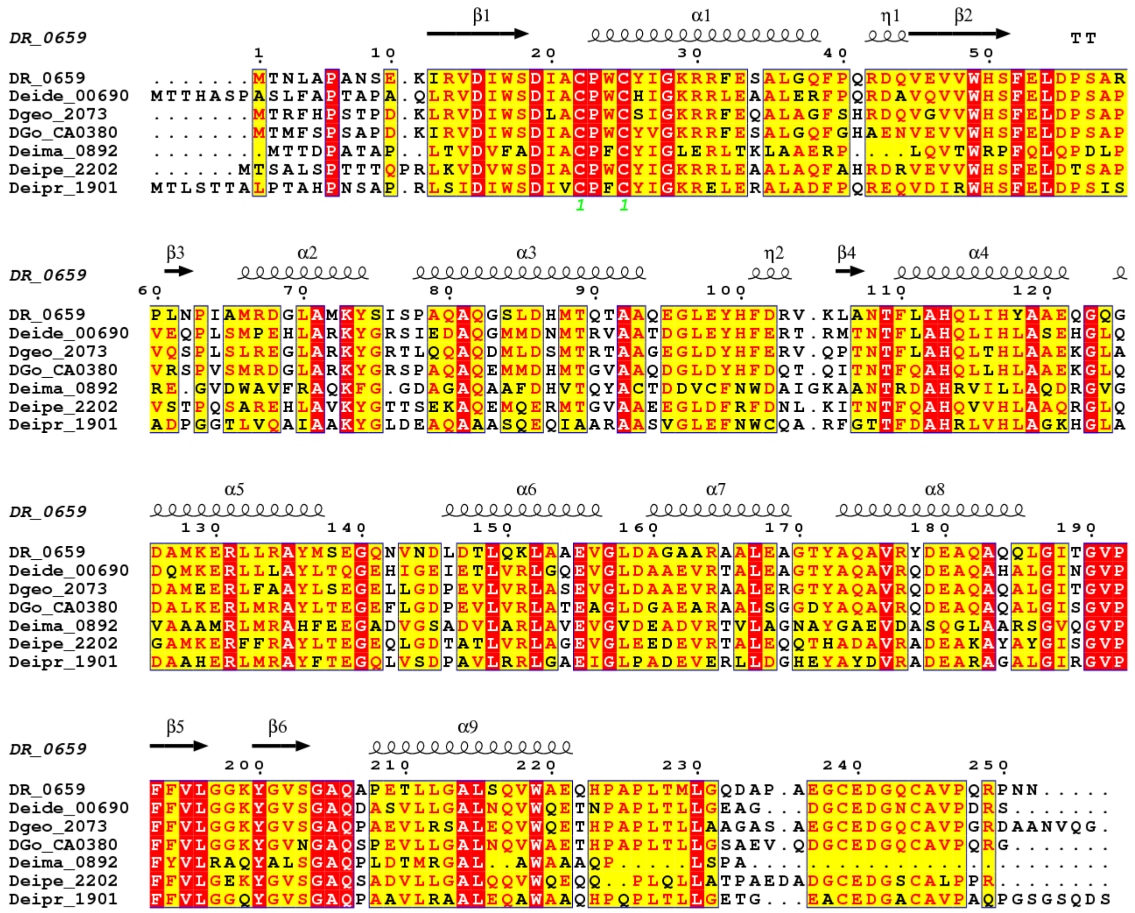

(b)

```
Deide_3p01230  MSDLHLLYVTDAYCGWCWGFAPTLSAFHARH-PHLPLRLISGGLFTGEKIAPIAAYPHIP  59
Deipr_1559     MTKTKLTYVTDTYCIWCWGFGEALRGFAQQNADRIELEVLPGGLLVGDRVQPVGEKPRVL  60
               *:.  :* ****:** *****.  :*  .*   ::   :: *.:: ***:.*::: *:.  *::

Deide_3p01230  GANDRITHLTGVTFGDAYQARLQEGILVLNSDDAAAGLAALRALAPDRALEAFHAIQHAF  119
Deipr_1559     ESAARVANMTGVPTGEGFRRSVEEGSTVLDSGVAARAYWALHSLAPSRGLDIAHALQHAW  120
                :   *::::***   *:.::   ::**   **:*.  **  .   **::***.*.*:   **:***:

Deide_3p01230  YMEGQSLSDPRTYRAVAQTLNLDPDAAEAAFHGPQARTEAAQDYQLARTLGVDSYPTLLA  179
Deipr_1559     YWDGLDLHDPAVIGGVARELGLDAAAAARALADPAAEIQALAGFERRQTLDISGYPTLLV  180
               *  :*  .*  **  .    .**:  *.**   **   *:  .*  *.  :*    .::    :**.:..*****.

Deide_3p01230  QQDGQRTVLARGAATVEQVETRLQRALNPATP------  211
Deipr_1559     HGPHGTQRIGGARATPAKLTAAFEQVLAGETVEEEDEE  218
               :         :.  .  **   ::  :  :::.*    *
```

(c)

```
DR_2335     MTPQPTDAQPTDAQPTDLYFDFLCPYAWRGVEMAHVLRG-----SGEGFRLRHFSLVQGN 55
Deide_22890 ------------MTSTDLFFDFICPYAWRGLELVNALRA-----EGETFRLRHFSLVQGN 43
DGo_CA0030  ---------MLASAMTDVYFDFLCPYAWRGLELANVLRALPGDAGGETFRLRHFSLAQGN 51
Deima_0620  ---------MTQTQPLQVYIDFLCPFAWRGVELALILRET----RGLNVQLRHYSLVQGN 47
Deipe_1019  ------------MQPSSVYFDFLCPYAWRGLELVS----Q----LGVRPQLKHFSLVQGN 40
                            .:::**:**:****:*:.           *   :*:*:**.***

DR_2335     HPQNK---DQETVQWWLTDQPLGAEGGSGYMKYQRPSLNAFLAAHAAARQGEEKSWAFAL 112
Deide_22890 HADNA---GQPEPRWWLTDQPPGEGTV-----SQQSSLAAFLAAGAAARQGEEAAWAFSL 95
DGo_CA0030  HPDNAAAKGAGDVRWWLSDQPQGEGAA-----HQQSSLDAFLAHTAAARQGEERSWAFAL 106
Deima_0620  HPENP---DRKQPTWWLTDQTADSGSD-----MQRGSLAAFLAAHAAARQGEQERFTFTV 99
Deipe_1019  HAQNP---DRKNPVWKLAAQPLTEGPD-----SQQASLRSFLAAQAARLQGNEAELQFTL 92
            * :*    .     * *: *             *: ** :***  **  **::    *::

DR_2335     ALFRLHHEDKRDLDEA-AFQDAATRAGLDLSQWKQDRQDEAGLRRELRADLEAAAALGVF 171
Deide_22890 ALLRIRHEDGQPLDEA-ALTQAAQTAGLDSGRWAADRADETGLREGLRTDLSEAHRLGVF 154
DGo_CA0030  ALFRRRHEHGQALDEA-AIQGAAGDAGLDTAQFAADRQDDGGLRAALRRELEEAADLGVF 165
Deima_0620  ELFRLRHQDGRALHDPTTLHAAAERAGLDAARFAQDLQDDAGLRAALTEDLRAAAALGVF 159
Deipe_1019  QLLRLRHDGRRDLNDPQTAREAANQAGLDTGRFETDLADEASLRESLARDLNDAAQLGVF 152
             *:* :*:  : *.:  :   **  **** .::  *  *: .**  *  :*  *  ****

DR_2335     GTPTFDLGGGDVAYFKFEELTRDPQAARDLWNLFTSTLRSEARVATIRRPVPKKG--- 226
Deide_22890 GTPTFVLPDGHAAYYRFDHLTRDPQVARERWQLYRDVLQSEAGIGTIKRTRRMTR--- 209
DGo_CA0030  GTPTFVLEDGGAAYYRFENLTRDPQTARAWWDLYRTVLDSGAGIATIKRARNRPAKKA 223
Deima_0620  GTPTFVLDGANAAYFRFARLPESPEAAHALWELYVQTLLNDARIETIKRPR------- 210
Deipe_1019  GTPTFVLPDGQAAYLRFSQLPADESAARRLWDTYVTVLTSDANIETIKRPR------- 203
            ***** * .. .** :* .*  . ..*:  *: :  .* . * : **:*
```

Figure S6. Multiple sequence alignment of *Deinococcus* FrnE reductases. **(a)** FrnE proteins (DsbA family, FrnE subfamily). The N-terminal CxxC corresponds to residues 22-25 in *D. radiodurans* FrnE (DR_0659), and the C-terminal CxxxxC to residues 239-244. **(b)** FrnE-like proteins found in two *Deinococcus* species (DsbA family, FrnE-like subfamily). The CxxC motif is highlighted in yellow. **(c)** Proteins containing a DSBA-like Trx domain. The single Cys is highlighted in yellow. Alignments were made as in Figures S1 & S5.

Figure S7

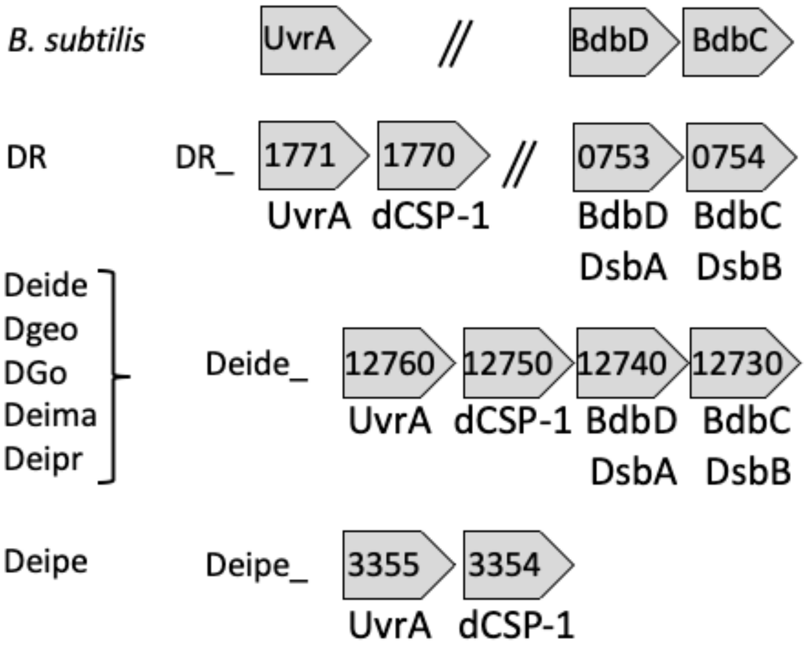

Figure S7. Putative operons encoding DsbA, DsbB and UvrA. Similar to the *B. subtilis bdbDC* operon encoding DsbA and DsbB homologs, putative operons encoding DsbA and DsbB family proteins are present in most *Deinococcus* species. In five of the seven analyzed species, these genes are likely in operon with genes encoding DNA repair protein UvrA and a membrane protein designated dCSP-1 (*Deinococcus*-specific conserved signature protein-1). In *D. radiodurans* (DR), the *uvrA-dCSP-1* genes are located separate from the *bdbDC* genes. *D. peraridilitoris* (Deipe) lacks the *bdbDC* homologs. Gene numbers (locus tags) for *D. radiodurans*, *D. deserti* and *D. peraridilitoris* are indicated.

Figure S8 (panels a to e)

(a) (DsbA family with N-terminal transmembrane helix)

```
DR_0753     -----MTRLSGNNQNRSVLIIGTLVAAALIALALFAVRGKSTTTT----GE--AQTFTYA 49
Deide_12740 -----MTRLQGSNPNRTMLVVGTLLAALLIGLALIAVRGKPAPGA----GL--TADFNLS 49
Dgeo_0692   -----MTRLQGSNQNRTVLVIGTLIAALLIALALFAARGRSSGQT----G---TANFDLT 48
DGo_CA1008  -----MTRLSGNNQNRSVLLIGTVIAVVLVALALFAVRGRPAGGA----ANTSTVNFNYA 51
Deima_1749  MSKPNLQRDGGSNANRMALLIGTVIAAVLIALALFTRHGNG---------NTQAQTFDLT 51
Deipr_0493  -----MTKLQGNRSNNMPLIIGTLIAAALIALALFAGRGKSGEQASTASGAAASAKFDLS 55
Deipr_2421  ----------MNRQNNRALLILTLALAVIAGLLMFNLAKRPKPQSSA---AISTEQLIRP 47
BDBD_BACSU  -----MKKKQQSSA-KFAVILTVVVVVLLAAIVIINNKTEQ--GN-----DAVSGQPSIK 47
                       .   .  ::: .:  . : .: ::    .             :

DR_0753     NLPYAGQANAPVNVLVVEDFKCPNCKSFEETVAPELRTKYVGTGKVKMYSLVYPFLAD-- 107
Deide_12740 TVPYAGLAEAPVSVVVVEDFKCPVCKTFEETIAPELTSKYVQTGKAKLYTVVWPFLAEAR 109
Dgeo_0692   GQPVLGQENAPVTLVVFEDFKCPNCKRFEEEFMPELRSKYIDTGKAKLISMNFPFIAAMS 108
DGo_CA1008  SLPYAGQESAPVNLLVVEDFKCPVCKQFEETVAPELRTKYVDSGQAKMYALVWPFLAQVV 111
Deima_1749  GRPVLGRADAPVTMIVFEDYKCPVCKGFDEEDLPTLKSKYIDTGKVKMVAMAYPFLAQNF 111
Deipr_0493  NKPVVGDASAPVEMIVVEDFKCPACKQFEATVFPKVENEYVSTGKVKVYSVAWPFLAEVA 115
Deipr_2421  DSPFLGPADAKVTIVEFFDPECESCAAVEPALMDVMQK-Y--NGEVRLVARYFPLHSN-- 102
BDBD_BACSU  GQPVLGKDDAPVTVVEFGDYKCPSCKVFNSDIFPKIQKDFIDKGDVKFSFVNVMFHGK-- 105
              *  *  .* * :: . * :*  *  .:      : . :  .*..:.     : .

DR_0753     RLPEDDSKYAAQAARCVYAQGKNDAFNTYKEILFRAQGPETEVWA-TKSRLKELATSL-D 165
Deide_12740 RLPTDDSKLAAQAARCVYDQGGNKAFGSFKSILFRAQGDEGTVWA-TKARLKELAANVEG 168
Dgeo_0692   NLPVDDSKLAAQAAECAYLQGGSEAYDRMKQILFRAQGAESEVWA-SKSRLKDLAGSVEG 167
DGo_CA1008  GLPTDDSKIAAQAGKCVYDQRGNDGFGAYKSILFRAQGDERTVWA-TKDRLKELAQNVEG 170
Deima_1749  GLSDDDSTRASVAAKCMARQG-TEKFWAYHHALFRGQQDEKTVWA-TEEALQDLAGTIDG 169
Deipr_0493  KLDEDDSKYAAQAGECAYEHGGAEAFSAYKTILFRAQEDESKVWA-TKARLKELAANVSG 174
Deipr_2421  ------STLAAGLI-EAAAQDSADKRWRMRDYLFQKQREWGEQQTAQTDKFLDYAED-MG 154
BDBD_BACSU  -----GSRLAALASEEVWKED-PDSFWDFHEKLFEKQPDTEQEWV-TPGLLGDLAKSTTK 158
                  *  *:        .   .     :  **. *       .     : : *

DR_0753     IDQAKFATCLDNDETAAQVETDKQEALKAGVGGTPTVFVNGKLVNVQS----DYVKDISA 221
Deide_12740 LDTGKFATCLDTDATASLVEAEKKMVEDARVNHTPTVFVNGKEVMNTQGQSSYLMADVSK 228
Dgeo_0692   IDQAKFNTCLDNDETAAAVEADKQQAEKAGVSGTPSVFVNGKLVSS------YDAATVGA 221
DGo_CA1008  LDQTAFATCLDSDATAARVDADEKQANDAKVSGTPTVFVNGKVVTGSNGQNSWQLADISA 230
Deima_1749  VDTAAFNTCLKDQATLKEVNDDKAQGDKAGVNGTPSVYVNGRYIANF------HADALGQ 223
Deipr_0493  IDQTAFASCLDNDETLARVEANEEEVEASGVTGTPTVFIGGKKVENPGDYG-----QLKS 229
Deipr_2421  LDRSKAQATMESAAVRDLLARDRKDGEAVGVTGTPTFFVNGKPLPELS------LEALEN 208
BDBD_BACSU  IKPETLKENLDKETFASQVEKDSDLNQKMNIQATPTIYVNDKVIKNFADYD-----EIKE 213
            :.       :.       :  :        :  **:.::..: :             :

DR_0753     AIDEALKP- 229
Deide_12740 AIEDASN-- 235
Dgeo_0692   AIDAASR-- 228
DGo_CA1008  AIDAAKQ-- 237
Deima_1749  AIEDAQK-- 230
Deipr_0493  AIDAALAN- 237
Deipr_2421  AIQEGLNE- 216
BDBD_BACSU  TIEKELKGK 222
            :*:
```

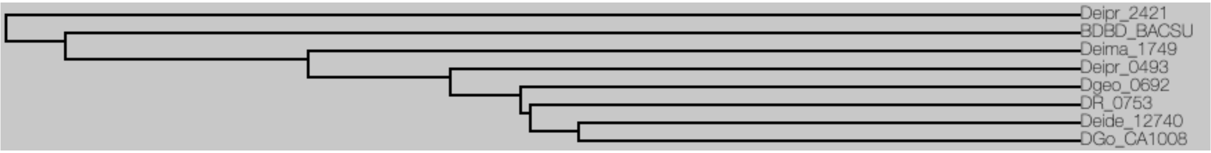

(b) (DsbB family, BdbC subfamily)

```
DR_0754      --MNRDTRLYLAWLVALAATLGSLYFSEIRHFNPCPLCWAQRIFMYPLAVILGIAAFVGD 58
Deide_12730 MLSSADNRIYAAWVVALVATLGSLYLSNVLGFKPCVLCWYQRICMYPLALWLGIAALRGD 60
Dgeo_0691    --MTRDNRLYLAWVVALLATLGSLWFSEVRQFVPCVLCWFQRIAMYPLALLLGIAALRAD 58
DGo_CA1007   --MSRDNRLYAAWVVALVATLGSLYFSEVRGFNPCVLCWYQRICMYPLALLLGVAAFRGD 58
Deima_1748   --MTRENRVYAAWVVAILATMGSLYFSEVRQFVPCVLCWFQRICMYPLVLVLGVAAFRAD 58
Deipr_0492   --MNRSTRLYLAWVVALVATLGSLYLSEVLGYRPCKLCWYQRIAMYPLALMLGIAAFRDD 58
BDBC_BACSU   -MKNRIVFLYASWVVALIAMLGSLYFSEIRKFIPCELCWYQRILMYPLVLILGIATFQGD 59
                .    :* :*:**: * :***::*::  : ** *** *** ****.: **:*::  *

DR_0754      HGVRRYVLPLAALGLGFAIFQNLETWG-FVQSIKACTVNAA-AACNTPWPVWGTS---Q- 112
Deide_12730 TGIRVYAWPLAAVGWIIALVQNAEDWG-WIPTLKACAPDPTTVACNIPWPLWGSG---AL 116
Dgeo_0691    LGIRVYALPLAAIGWVIALIQNLEDWG-VIPTLRVCSARAA-APCDVHWPVWGAG---A- 112
DGo_CA1007   LGIRAYALPLAVVGWLTALYQNAETWG-WVPVLKACTTNPA-ASCGTPWPVWGVG---N- 112
Deima_1748   PGGRAYALPLAVAGWLVAMVHNLEDWG-VIQALKVCGVGQTTAGCDAKWPIFGDA----N 113
Deipr_0492   LRVRLYAAALALIGAITALVQNAEIWG-WIPTLKSCSIDAGQEPCTTIWPLWSTLFGEGA 117
BDBC_BACSU   TRVKKYVLPMAIIGAFISIMHYLEQKVPGFSGIKPCVS---GVPCSGQYINW-------- 108
                : *.  :*  *   :: :  *     .  :: *        *   :  :

DR_0754      DTLNRALTIPVLSMIAFALILALLSWPRQRVTVPESAAVQG----- 153
Deide_12730 SGLNSVITIPVLSMIAFTLILALLSWRRERFI-------------- 148
Dgeo_0691    -SLNSVLTIPVLSLVAFTLIIGLLSWARERKV-------------- 143
DGo_CA1007   DALNTTITIPVLSMIAFTLIIALLSWRRVARTTADQTVAAQA---- 154
Deima_1748   KAVSDIITIPVLSFVAFTLVIALLTWRERPGRR------------- 146
Deipr_0492   SALNSILTIPVLSMIAFSLILALLAWPRMRPEEQAQATYTEPHDRV 163
BDBC_BACSU   ---FGFITIPFLALIAFILIIIFMCLLKGEKSE------------- 138
                   :***.*:::** *:: ::   .
```

(c) (DsbA family with N-terminal signal peptide)

```
DR_2019     MPSFLSFLSGRGPLVAAALLALSGAPAQAQLWETPQTT-ARQPLLRGVSVQPGGKVLRLG 59
DR_0560     MLW------AMKRLLAALLGALLLPSAHAQLG-QPTDALLKSPLFVGAQTSRAGIITLKS 53
Deide_06420 MNK---------VLLGLVWGVALAPVASAQLWETPQAT-VRQEVLKDFSV--AGNVLQGP 48
Dgeo_0747   MNRVIG------LVAGALLAANLGSQASAQLLATPAAT-AAQPLLAGFRV--SGSTLTRG 51
DGo_CA1399  MTH------FARRFFALGLLAALSGAAHAQVG-GQVAPVRAAPAFQGAKVGAGGLLTLTD 53
Deima_1134  MNRTL---------LTAALSVLG--VAAAQV-AYPASNYARLTGGTAASATASTTSVRVG 48
Deipr_0943  MK----------PHMSFLVAALLLPGAQAQLWNTPQAT-ASQPLLRSFKA--EGDVLTHG 47
            *                   .     * **:                  .

DR_2019     STTLTLDVVAGRVVGVLIEG--RDTASVARA----------------------LAAVWDS 95
DR_0560     GVNVVLGQRGGRLTTVTITTPYTEPQGISGSSDTAVAAGSVEQRVGGSALAAQTVGAVTG 113
Deide_06420 ETRVTLDVSGGRVVGVLVEA--ATGTALARG----------------------VAAAWGM 84
Dgeo_0747   TTTLTLDTAGGRVVGVLVQA--DNPQDVARA----------------------LVAAWGG 87
DGo_CA1399  GAQVTLSQKAGYLAGASVSV-----------------------PASGAARAAELLGVLSG 90
Deima_1134  GGTVTVTTAGGLAARFAYSGPTDDTASAARA----------------------LIATETD 86
Deipr_0943  QTRAELDRIGQRVIGVYVRAPKANTEDVARA----------------------ILSAWGA 85
                 :   .                                             ..

DR_2019     TAEGAA-----SLQQAFARPAFQDRARLGSVQPSDENGTDLLAIRLTGRGAEQRWRVYA- 149
DR_0560     YGEGLS----APLLQFLRQPDV---VKQLPQGVTVDAPPLTIQAQVQGRALVLK-----L 161
Deide_06420 READLP-----QLVRQLSSSELLASARRGFTEFTDESEQDMITVKVTGQGAQVRWRAYL- 138
Dgeo_0747   PEQNVA-----AVAQALGRDDL-QREARGAQGLRDDSEGTLLRVKLNGTGGAQRWTAYT- 140
DGo_CA1399  YEDGLA----EPLEAFLKRSDV---AGRLPQGVTVDAEPFTVTARLTGGRLNVS-----L 138
Deima_1134  MDAPVTAAQAKQLAGAFDKIR----AQVLGKGPVPLG-------FADGLDFTLDWTASRL 135
Deipr_0943  PEDAVP-----DLKGVLDDPGFQQQAQQLFEEVSEDGGSAV-YVRL----QDGTWQAYT- 134
                        :   :

DR_2019     AVNVLPE--SDFPVTRNVT---GQSKAPNVIHVLSDFQCPACRQLWAEQ-IPGWRA---Q 200
DR_0560     SMTQVPA--GQFTATKNLRPAA-KPGKDVVLRVYSDFQCPYCQKLELET-MPALLRA--L 215
Deide_06420 ALKIWPD--AAFPATRNVS---GRADAPNSIRIFSDFQCPYCRDLWHET-LPGWAR---Q 189
Dgeo_0747   ALLIFPD--RAFPATANVQ---GSAQAPNVLRIFSDFQCPYCKELWDTA-HPKWAA---Q 191
DGo_CA1399  VLAQVPA--AQFAPVKTAQAARTATASPVVLRVYSDFQCPYCQQFETQT-LPELQKA--L 193
Deima_1134  TFTVAPHEYTGFGADRYVL---GKG--GPVIREFSDFQCPYCRELHDDV-FPALQRDLIG 189
Deipr_0943  ALKVYPH--SAFPQVSAPL---GNDGAPARLNIVSDYQCPYCNQLWNSASMAEWRS---K 186
             .   *     *                  :.  **:*** *..:

DR_2019     PAKYRLFYHHFPL-SYHANAFAAAEASECAAKQGQFWTYADRLFGGVEEWGRASAPQATR 259
DR_0560     PDDVRVEFHQFPLEQIHPLARPAAEASECAAQQGRFWDYKDALFRDRSWLQ----NNPNE 271
Deide_06420 TTQYRVAHYHFPL-DFHKNAFAAAEASECAAAQGAFWKMADQIFAGFDVWNRLSARDAAT 248
Dgeo_0747   PNVYRVMHYHFPL-SFHKNAEPAAIASECAAEQGKFWPYADLLFRHTAEWTGLPS--ASA 248
DGo_CA1399  PADVRIEFHQFPLESIHPRARAAAEASECASAQGKFWAYKDALFADRSWLS----GDAAT 249
Deima_1134  KGLARFSYRHFPL-SFHQNAMPLALGGECAAQQGKFWAYHDVAFTVTSP----------- 237
Deipr_0943  PGVYRLNYHHFPL-SFHPLALPAAQFSECAAQQGRFWEFSDAVNADFAHWTQQPEAEARQ 245
                *. . :*** . *  *   *  .***: ** **   *

DR_2019     LFGTYAGKLKLDRTAFDRCLNTHQLKAKVQRQIQGAGKTYLRGTPTVYLNGVKLNSFSDE 319
DR_0560     TFLRLAGDLKLDPGKFKDCLALRGGKAGVDAGLAEAQQLGLNATPTVFVDGYRVGNPFDT 331
Deide_06420 QFRTYAGNAKLTPATFEKCMTQRSSRAVVDAQIKAGLTLGVKGTPTVFLNGMKLQNYTDA 308
Dgeo_0747   KFSEYAQAAGLNVAAFQTCLTSAAPKAVVRAQQAAGLKLGVQGTPTVYLNGVQLRNYSDE 308
DGo_CA1399  AFTAIAGKAGLNLTAFKGCLAARGGRAAVDAGLAEADRLGLQGTPSVFVNGYAVADPYDA 309
Deima_1134  --VTAAKQLGLNLTTFQTCLKDPAVQALVKADMKVGDAVDVQGTPSLYVGPFKVQNWTDA 295
Deipr_0943  SFTRYAVSAGVTQAELDKCLAQDRSRDIMATADQLQRQLNVRGTPSVYLNGIKLNNYNDA 305
                 *    :    :. *:     :  :           :..**::::.   : .  *

DR_2019     -ELASVQAVTRAQPSAASV---IAA--RFKSFR---------- 346
DR_0560     AAVLQLIDVARATR----------------------------- 345
Deide_06420 SEWAQVQAVTTAKPSAAQL---IES--RLAQFR---------- 336
Dgeo_0747   NELAAVRAVTAASPGAVEV---IAA--RLKRF----------- 335
DGo_CA1399  PALLRLIAYARAVDTAPAAATPVTAPSPLPSPKAVPATPPATR 352
Deima_1134  ASIGNYVKLVTALGK---------------------------- 310
Deipr_0943  AQIRAIRAVTEAGPGAEKV---IEQ--RLKGLR---------- 333
                     . *
```

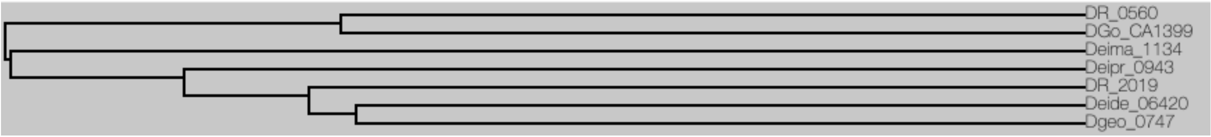

(d) (Cytochrome c biogenesis protein CcdA, DsbD family)

```
DR_1300        MLPGVNVSAAPSLTVAFLAGLISFLSPCVLPLLPSYLGVLGGA-------------KAPL 47
Deide_08350    ----MMSTGAPTLTVAFLAGLVSFLSPCVLPLVPSYLGAIGGT-------------RAPL 43
Dgeo_1241      ---MFASPGAPSVTVAFLAGLISFLSPCVLPLVPSYLGVLGGA-------------RAPL 44
DGo_CA1639     --MLAQALAAPTLAVAFVAGLVSFLSPCVLPLVPSYLGVIGGA-------------RAPL 45
Deima_1155     ------MSSAPTVTIAFLAGLVSFLSPCVLPLVPSYLGVLGGA-------------RAPM 41
Deipe_0794     -MNPAQAEVLPALGIAFAAGLLSFLSPCVLPLVPSYLGVLGGG-------------RSPL 46
Deipr_0982     ---MVEAASAPTLTVAFVAGLLSFLSPCVLPLVPSYLGVIGGT-------------RAPL 44
Deide_2p00430 ------MTDSPGFLFAFGAGLLSFLSPCVLPMLPAYLGFLTGMSRGELAGPQ--ARRLAL 52
Deipe_0661     ------MISSPSFLFAFGAGLLSFLSPCVLPLLPAYLGFLTGMSRGELSERA--ARRPAL 52
CCDA_BACSU     -------MGDVNYFLTFGAGFLSFISPCCLPLYPAFLSYITGVSMDDVKTEKLLLQKRSL 53
                             .:* **::**:*** **: *::*. : *              :  :

DR_1300        GRALGFIAGFGLVFVALGATASTLGAFLAPHKLLLGQLAAVLIVFFGLVMLGVIRLPFLM 107
Deide_08350    ARALGFIAGFGLVFIALGATASTLGSVLAPHKILLGQVAGGLIVFFGLVMLGLIRVPLLM 103
Dgeo_1241      LRALGFILGFGLVFIALGATASSLGALLAPHKLLLGRVAAVLIIFFGLVMLGVIRLPWLM 104
DGo_CA1639     LRAAGFILGFGLVFMALGATASTLGAALAPHKALLAQLAGVLILFFGLVMLGALRLPFLM 105
Deima_1155     TRALGFVLGFGLVFVALGATASSLGALLLQYKYLIGRVAAVLIILFGLVMLGVIRLPFLM 101
Deipe_0794     VRALGFVMGFGLVFIALGATASYLGALLAPHKIVLGRAGAILIIAFGLFMLGLRPRGLM- 105
Deipr_0982     VRALGFIAGFGLVFVALGATASALGALLAPHKLLLGRLAGALIVFFGLVMLGVIRLPWMM 104
Deide_2p00430 SHAVAFLAGFSVVFLALGALVESAAVVLTTYGTPVRVIGGAMVLMMGLFTLGVLRVNALY 112
Deipe_0661     KHALAFLAGFGVVFLALGALVESASIILTTYGTPVRVLGGVIVLLLGLVTLGALRAPWLK 112
CCDA_BACSU     FHTLCFLLGFSVIFIALGYGTSFIGSLFRDYHDAIRQIGALLIILFGFITLGVFRPEAMM 113
                 ::  *: **.::*:***  ..  .  :  :   :   .. ::: :*:. **      :

DR_1300        RDTRQ-LA--DAGGYGPVALGAAFAFGWSPCLGPTLGSILGLAASSASLGSGVRLLVAYT 164
Deide_08350    RDTRA-LA--GAGSYSPVALGAAFAFGWSPCLGPALGSILGLAASSASLSSGVVLLAAYT 160
Dgeo_1241      RDTRA-LA--DAGGYGPVALGAAFAFGWSPCLGPALGSILGLAASSASLSTGVGLLAAYT 161
DGo_CA1639     RDTRQ-LA--DAGGYGPVALGAAFAFGWSPCLGPALGSILGLAASTASLQTGVGLLAAYT 162
Deima_1155     RDTRTALY--GADRYGAVALGAAFAFGWSPCLGPVLGSILGLAASGASLPLGVGLLAAYT 159
Deipe_0794     LDSRR-MR--GAERYGAVVLGAAFAFGWSPCLGPILGSILSLAASSANLPRGVSLLGAYT 162
Deipr_0982     RDTRQ-LS--AADRYGPVALGAAFAFGWSPCLGPALGSILGLAASTSSLSQGVVLLLTYT 161
Deide_2p00430 LERRVQLKSKPAGYLGSAVVGLAFAAGWTPCMGPILAGVLFMAAQQP--ALGVPLLLTYA 170
Deipe_0661     MERRTHLARKPAGYLGSALVGLAFAAGWTPCMGPILAGVLFVAAQQP--GLGVPLLLTYA 170
CCDA_BACSU     KERRIHFKHKPSGFLGSVLIGMAFAAGWTPCTGPILAAVITLAGTNP--GSAVPYMMLYV 171
                : *  :    :    . . :* *** **:** ** *..:: :*.       .*  :  *.

DR_1300        LGLALPFLLAALLWDRLNLRRLNRYAGIFEKVGGAVLVIVGVMMLTGQFTRLATFFFSVM 224
Deide_08350    LGLAVPFLLAALLWHRLNLRRLNRYSPVFEKVGGVVLVAMGLLMLSGEFTRLASFFFTIM 220
Dgeo_1241      LGLAVPFLLAALLWHRLNLRRLNRYAGVFEKVGGAVLVLVGMLMLTGQFTRLATFFYEVM 221
DGo_CA1639     AGLALPFLLAALLWDRLKLRRLNRYAGVFEKVGGALLVVVVGVLMLTGQFTRLASFFYSVM 222
Deima_1155     LGLAVPFLLAGLLWNRLNLRALNKYSPVFEKIGGALLVLVGTLIVTGAFTRLNSFFFEVT 219
Deipe_0794     VGLAVPFLLAALLWRRLNLRALSRLSPVFEKIGGAILVVVGLLIFSGEFTRLAGFFFEIT 222
Deipr_0982     LGLAAPFLLAALLWDRINLRGLNRYAGIFEKVGGAILVAVGLLMLSGYFTVLAAFFYSVM 221
Deide_2p00430 LGFSVPFLLGALFLERV--RALHRFTPRLERAGGALMVVAGILLITNGFAWISRSLVNV- 227
Deipe_0661     LGFSVPFLLGALFLERI--RLLGRFTPTLERVGGVMMVVAGVLLLTNGFAVMSQYLVNV- 227
CCDA_BACSU     LGFAVPFLLLSFFITKL--KWIRKNQLFIMKAGGVLMIVIGVLLFFNWMSLIIILLSDLF 229
                *:: **** .::  ::  : : :    : : **.:::  * ::. . :: :   :  :

DR_1300        PEWLKV-- 230
Deide_08350    PDWMRI-- 226
Dgeo_1241      PAWLRV-- 227
DGo_CA1639     PAWLIS-- 228
Deima_1155     PAWLRL-- 225
Deipe_0794     PAWLRQYL 230
Deipr_0982     PQWLRV-- 227
Deide_2p00430 VGFQGF-- 233
Deipe_0661     LGFQGF-- 233
CCDA_BACSU     GGFTGF-- 235
                :
```

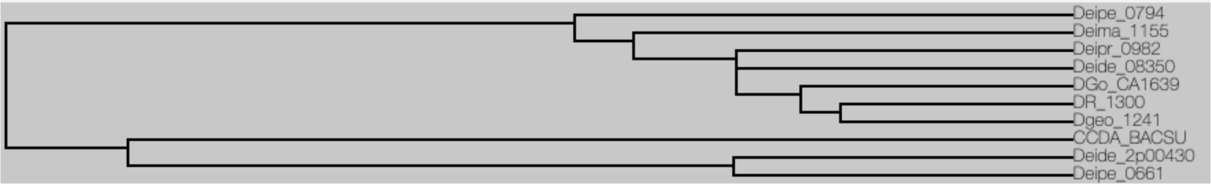

(e) (DsbE/CcmG homologs, TlpA-like family)

```
DR_0189       MTEPA--------PASPARPAWTRALPPLLAAALVGGLGWALLKPAG-NA--A-NGPLVG 48
DR_0345       MTSPTPPT-SPSLSPQPRRASWTRWIVPAVMVGLVGLLAYGLFTPDP-EG----GPALLG 54
Deide_08290   MTQ----PAASS-TNSAPVPAWKRLLPPLIAAGLVGVLGVALFRPAD-DA--TTGGPLVG 52
Dgeo_1248     MTE----LPSTS-KPAAPAPLWRRLIPPLLAAALVAVLGIALLNPAH-NA--TDGGPLVG 52
DGo_CA2017    MTE----KTAPSNPAPAARPSWRRFVPPLLAAGLVAVLGVTLLRPAR-TA--TDGGPLIG 53
DGo_PC0193    -----------M-TPDRPPPLWKRLLPPLLAAGLATSLAVALLNPAR-NA--TSGGPLVG 45
Deima_1148    --------MTTSPSSPPAVPTWRRLLPPALAFLLVVVFAVALTRQSD-SARTGATGPLVG 51
Deipe_0801    ---------------------MKRWIPPILAGLLVIVLAAALLRGSG-----DATGPLVG 34
Deipe_4366    ------------MPRSPVRCLLRRWIPPILSALLVIVLAAALLRGGA-----E-GVPLLG 42
Deipr_0892    MTRPAPHDPADLQPTSPSGPAWKKWLPPLLAFGLVAALGTALFSPTR-NE--TAGGPLVD 57
Deide_2p00420 ----------------------MRLL-RIKGRTIQKQLTLLLFLITS-L----ASAVRPG 32
Deipe_0660    ----------------------------------MRVLTTLLLLSLG-V----AQAVRPG 21
YNEN_BACSU    --------------------MLKKWLAGILLIMLVGYTGWNLYQTYS-KK---EVGIQEG 36
RESA_BACSU    -----------------MKKKRRLFIRTGILLVLICALGYTIYNAVF-AG---KESISEG 39
DSBE_ECOLI    ------------------MKRKVLLIPLIIFLAIAAALLWQLARNAEGDDPTNLESALIG 42
                                                       :                 .

DR_0189       KPAPQFNLTGLDGQPVALA---DYRGRPVVLNFWASWCGPCREEAPLFAKLAAHP----- 100
DR_0345       KPAPAFALEDLGGRTHALT---AAQGKPVVINFWASWCVPCRQEAPLFSKLSQET----A 107
Deide_08290   KTAPDFILQSLDGPELRLS---SLKGRPVVVNFWASWCVPCREEAPLFRDLSARQ--APG 107
Dgeo_1248     KPAPEFTLTSLDGTPVSLA---SLRGRPVVLNFWASWCGPCREEAPLFRELSTRQ--SAG 107
DGo_CA2017    KAAPEFDLQTLDGGTLSLA---SLKGRPVVVNFWASWCVPCRQEAPLFRELGARPANAGG 110
DGo_PC0193    RPAPDFTLESLDGVDVRLA---ALKGRPVVLNFWASWCAPCREEAPLFRELSERQ--TAG 100
Deima_1148    KAAPNFTLKDLNGNTVTLA---SLRGRPVLLNFWASWCPPCRNEAPLLSDVARQQ---RA 105
Deipe_0801    KTAPDFHLQTLDGGDVRLS---DLRGRPVIVNFWASWCIPCREEAPLLREIAEQQ---AE 88
Deipe_4366    QAAPNFKLETLDGSSLEFA---QLRGRPVLINFWASWCLPCREEAPLLQELAARQ---DD 96
Deipr_0892    KAAPDFRLTSLDGTPVSLS---DFRGRPVVLNFWASWCGPCREEAPLFRELSERQ---GA 111
Deide_2p00420 DVAPDFTLNDPSGKKVTLS---ALRGQPVVLTFWATWCLVCKEELPELNQEAARA----- 84
Deipe_0660    DVAPNFSLKTEAGKTVSLN---SLRGEPVVLTFWATWCLVCKEELPELNQEARAA----- 73
YNEN_BACSU    QQAPDFSLKTLSGEKSSLQ---DAKGKKVLLNFWATWCKPCRQEMPAMEKLQKEY----A 89
RESA_BACSU    SDAPNFVLEDTNGKRIELS---DLKGKGVFLNFWGTWCEPCKKEFPYMANQYKHF---KS 93
DSBE_ECOLI    KPVPKFRLESLDNPGQFYQADVLTQGKPVLLNVWATWCPTCRAEHQYLNQLSA------- 95
                .* * *    .           :*. *.:..*.:**  *: *   : .

DR_0189       GAPAVLGILF--NETKPQNARDFARQYGL--TYP-NLQDP-------GVATAIAYQVTGI 148
DR_0345       GKAEFFGVIY--NDQP-ADARRFMDQYGL--IYP-ALLDP-------GSRTALSYGVGKL 154
Deide_08290   TGLAVVGVLF--QEPREDAARTFIQEYAL--AYP-NLRDP-------KARTAINYGVAGI 155
Dgeo_1248     QGLAVVGILF--QETNEQNARDFIREYAL--AYP-SLRDP-------GIQTGINYGVSGI 155
DGo_CA2017    QGLAIVGVLF--NETREQDARDFIREYAL--AYP-NLRDP-------GISTGINYGVSGV 158
DGo_PC0193    RGLAVIGILF--QEPKEANARTFIREFSL--AYP-NLRDP-------KSATAINYGVAGI 148
Deima_1148    QGLAIVGVIY--ADDNVSALRDFIGEYNL--AYP-NVRDP-------GSRIAIDYGVAAV 153
Deipe_0801    GGLVIVGIMY--QDRE-ADARKFIEDYGL--TFP-SLIDK-------DLSTAIDYGVGAV 135
Deipe_4366    RGLVVVGVVF--QDTL-KNARAFRDEFNL--TFP-SVFDP-------GSRTAIDYGVSAI 143
Deipr_0892    GGLAVVGILF--EEKNEQNARDFIREYAL--AYP-NLRDQ-------NLNTAIDYGVGAI 159
Deide_2p00420 KVKNMFAV---SATDTPKAALDYFKQAKLGAITP-LVDAPKAKGPGTGAGVAKSYRIIGQ 140
Deipe_0660    KLKNMYAV---SATDSAKDALAYFRKNELGSVTP-LVDAPGTKGTSTAASVARAYRIIGQ 129
YNEN_BACSU    DKLAVVAVNFTSAEKSEKQVRAFADTYDL--TFP-ILIDK-------KG-INADYNVMSY 138
RESA_BACSU    QGVEIVAVNV---GESKIAVHNFMKSYGV--NFP-VVLDT-------DRQVLDAYDVSPL 140
DSBE_ECOLI    QGIRVVGMNY---KDDRQKAISWLKELGN--PYALSLFDG-------DGMLGLDLGVYGA 143
                  . .:              :             :                   :

DR_0189       PRTVFIDAQGVVRHIDQGGLDTARLNAGLSKIGVPGL----- 185
DR_0345       PITFIVDGQGKVVHIKDGPIEEPELRAALKQAGL-------- 188
Deide_08290   PETVFIDAKGVIQHVDRGGLDRARLNVGLEKIGVKGL----- 192
Dgeo_1248     PETFFIDREGVIRHVDRGGLTRERLNVGLKKIGVPEL----- 192
DGo_CA2017    PETVFIDKNGVVQHMDRGGLDRARLNVGLAKIGVPGL----- 195
DGo_PC0193    PETVFIDRQGVIQFMDRGGLTRERLNMGLETIGVPGL----- 185
Deima_1148    PETFFIDKTGVIRAHVRQEVTRDVLTRQLKTIGVSQ------ 189
Deipe_0801    PETFLIDRDGVVQRHLRQPLTRELLRDELPKIGVRL------ 171
Deipe_4366    PETFFVDRHGVVRAFVQGTLTREHLTRELPKIGVTP------ 179
Deipr_0892    PETFFIDKDGVIRYKDKGGLDRARLNAGLKTIGVEPL----- 196
Deide_2p00420 PVSVFIDSKGKVTAVHSGYLPPEQFRVYLKQIRP-------- 174
Deipe_0660    PVSVFIDKAGKVSAVHSGYMPPEQFRVYLKTIQTP------- 164
YNEN_BACSU    PTTYILDEKGVIQDIHVGTMTKKEMEQKLDLD---------- 170
RESA_BACSU    PTTFLINPEGKVVKVVTGTMTESMIHDYMNLIKPGETSG--- 179
DSBE_ECOLI    PETFLIDGNGIIRYRHAGDLNPRVWEEEIKPLWEKYSKEAAQ 185
              * : :::  * :       :        :
```

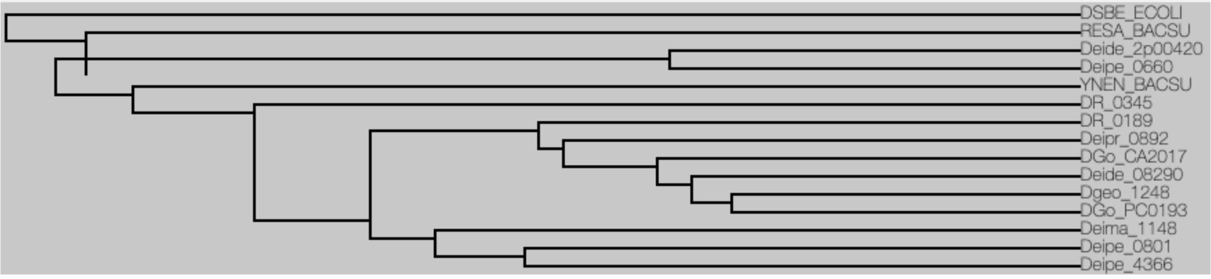

Figure S8. Multiple sequence alignments of *Deinococcus* Dsb oxidoreductases. **(a)** DsbA family proteins with an N-terminal transmembrane helix from *Deinococcus* species aligned with *B. subtilis* BdbD. Except for the second homolog of *D. proteolyticus* (Deipr_2421), the genes are adjacent to a gene encoding the DsbB/BdbC homologs shown in panel **(b)** (see also Figure

S7). Residues highlighted in cyan in Deipr_2421 differ in the proximity of the active site. **(c)** DsbA family proteins with N-terminal signal peptide. **(d)** Homologs of the DsbD family protein CcdA from *Deinococcus* aligned with *B. subtilis* CcdA. The deinococcal *ccdA* homologs are in gene clusters also encoding DsbE/CcmG homologs (see Figure S9). **(e)** DsbE/CcmG homologs from *Deinococcus* encoded by gene clusters also encoding CcdA and by isolated genes aligned with the TlpA-like family proteins ResA and YneN from *B. subtilis* and DsbE/CcmG from *E. coli*. Cys residues and CxxC motifs are highlighted in yellow. Guide trees showing sequence relationship between various proteins are included in case more than one homolog is present in the same *Deinococcus* species. Alignments and trees were obtained with UniProt ClustalO.

Figure S9

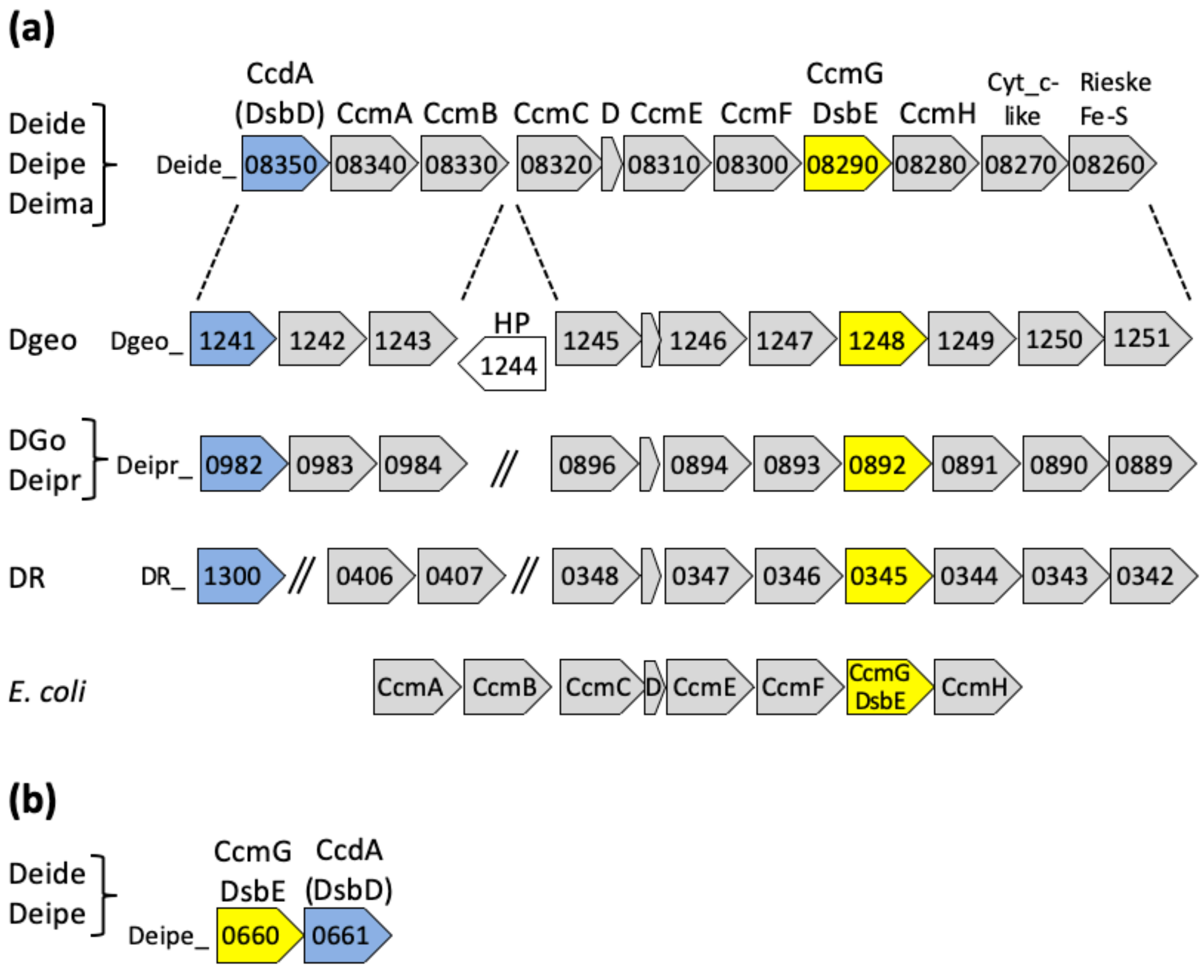

Figure S9. Gene clusters encoding CcdA and DsbE/CcmG homologs. **(a)** The seven analyzed *Deinococcus* species possess *ccdA* (DsbD family) and *dsbE*/*ccmG* homologs in gene clusters containing other cytochrome c biogenesis genes. Different genetic organizations are present in the different species, as shown. The *ccm* cluster from *E. coli* is also shown for comparison. Gene numbers (locus tags) for the indicated *Deinococcus* species are indicated. HP, hypothetical protein. **(b)** Additional two-gene cluster encoding CcdA and DsbE/CcmG found in two *Deinococcus* species.

Figure S10 (panels a & b)

(a)

```
DR_1849     ---------------------------MTQQTNSQGTQPGAAQEQAIFAGGCFWCTEAVM 33
Deide_10980 ----------------------------------MTDTAMQGMQQAILAGGCFWCTEAVM 26
DGo_CA1541  -----------------------------------MTNGTQGKEQAIFAGGCFWCTEAVL 25
Dgeo_0843   ----------------------------------MTSAQSEQTQQAIFAGGCFWCTEAVM 26
Deima_1788  ------------------------------------------MNQVILASGCFWCTEAVF 18
Deipe_3499  ----------------------------------------MAQEIATLAGGCFWCTEAVF 20
Deipr_1412  ------------------------------------MTQAHPSEQAIFAGGCFWCTEAVM 24
MSRA_ECOLI  MSLFDKKHLVSPADALPGRNTPMPVATLHAVNGHSMTNVPDGMEIAIFAMGCFWGVERLF 60
MSRA_BACSU  --------------------------------------MSEKKEIATFAGGCFWCMVKPF 22
                                                       : . :* ****     :

DR_1849     QDLRGVQKVESGYIGGTVPNPDYRSVCGGQTGHAEAVRVTFDPNQISYRDLLGLFFATHD 93
Deide_10980 KDLRGVHKVESGYIGGHTARPDYRSVCSGTTGYAEAVRVTFDPAQVSFRDLLGLFFATHD 86
DGo_CA1541  KDIQGVDKVESGYIGGDVPDPTYRAVCGGDTGHAEAVRVTFDPAQVSYKDLLGLFMATHD 85
Dgeo_0843   KDVRGVTRVESGYIGGHVPNPDYAAVCSGETGHAEAVRVTFDPAQVSFRDLLMLFFATHD 86
Deima_1788  KNVRGVQRVESGYIGGHVPNPTYNQVCGGDTGHAEAVRLTYDPNVISSRDLLGIFFATHD 78
Deipe_3499  QEVLGVSSVESGYIGGADPNPTYEQVCGGRTGHAEAVRITYDSQVISYEDILGIFFATHD 80
Deipr_1412  LRVRGVQQVESGYIGGRRPNPSYEQVCTGVTGHAEAVRVTFDPAEVSYRDLLHIFFGTHD 84
MSRA_ECOLI  WQLPGVYSTAAGYTGGYTPNPTYREVCSGDTGHAEAVRIVYDPSVISYEQLLQVFWENHD 120
MSRA_BACSU  DEQPGIEKVVSGYTGGHTENPTYEEVCSETTGHREAVQITFHPDVFPYEKLLELFWQQID 82
                *:  . :** **    * *  **   **: ***::.:.   .  ..:* :*    *

DR_1849     PTSLNRQGADVGTQYRSALFPLTQEQEQTAREMIEQLGTED---VFGRPIVTSIEPASTF 150
Deide_10980 PTTLNRQGADVGTQYRSAVFPLTPEQERETREMIADLNAQN---IFEAPIVTTIEPASEF 143
DGo_CA1541  PTSLNRQGADVGTQYRSAVFPLSAEQERETREVFADLGAQN---VFDRPIVTTIEPASEF 142
Dgeo_0843   PTSLNRQGADVGTQYRSAVFPLNEEQERETREVIGELTAQG---VFERPIVTTIEPAGPF 143
Deima_1788  PTQLNRQGADVGTQYRSAVFYANDEERQTAQAVIDELNAGN---VFDAPVVTTLEPATTF 135
Deipe_3499  PTTLNRQGGDVGTQYRSAIFAHGDSQRVTAERVIAELNDAH---IWEAPIVTTIESDGPF 137
Deipr_1412  PTTLNRQGADRGTQYRSALFPLTDAQRAEAQAVIEELNTS----TFGGAIVTSIEEPSEF 140
MSRA_ECOLI  PAQGMRQGNDHGTQYRSAIYPLTPEQDAAARASLERFQAAMLAADDDRHITTEIANATPF 180
MSRA_BACSU  PTDAGGQFADRGSSYRAAIFYHNDKQKELAEASKQRLAESG---IFKDPIVTDILKAEPF 139
            *:    *  * *:.**:*::     :   :.     :            :.* :     *

DR_1849     YVAEDYHQNYYKNNPGQGYCMA-V---ISPKVAKLRQYYGDKLR----- 190
Deide_10980 FVAEAYHQDYYANNPNDGYCRA-V---IAPKVAKLRKYYGEKLRA---- 184
DGo_CA1541  YVAEDYHQDYFANNPRQPYCAA-V---IAPKVAKLRKYYGDRLKAHARA 187
Dgeo_0843   YVAEDYHQDYYARNPYQPYCMA-V---ITPKVAKFRKAYSDRLRG---- 184
Deima_1788  YVAEGYHQDYYERNPGQPYCMA-V---ITPKVIKFRKQFSSYLS----- 175
Deipe_3499  YKAEPYHQDFFRRNPGQGYCLA-V---VAPKVVKFRRQFAQRLKSA--- 179
Deipr_1412  YVAEEYHQDYYANNPQNPYCSA-V---VGPKVAKLRQSYARFLNE---- 181
MSRA_ECOLI  YYAEDDHQQYLHKNPYG-YCGIGGIGVCLPPEA---------------- 212
MSRA_BACSU  YEAEGYHQHFYKKNPAHYQRYRTGSG----RAGFISEHWGAK------- 177
            : **  **.:  .**
```

(b)

```
DR_1378     ----MTQDTKTDFQKPSDNDLRERLTPIQYQVTQHEGTERAFTGEYWDHDEDGIYVDVVS 56
Deide_04050 -------MSKADFRKPADAELRERLTPIQYQVTQHEGTERAYTGEYWDHTEEGIYVDVVS 53
DGo_CA0919  ---MTQSPPKKTYSKPSDSELRERLSPIQYQVTQHEGTERAFTGEYWDTDEDGIYVDVVS 57
Dgeo_2072   --------MTRDFVKPSEAELRQKLTPEQYRVTQQEGTERAFTGEYWDHDEPGIYVDVVS 52
Deima_1441  ---------MSEYVKPTDAELRERLTPQQYAVTQHEATERAFTGEFWDHEEPGIYVDVVS 51
Deipe_4299  MTNSTDNDTFLTGLPSTEAEWRERLSPEQFRVLRQAGTERAFTGEYVDTDEEGSYHCAAC 60
Deipr_1900  --MTKPNWTPEGYRKPADADLRAQLTPEQYQVTQHEGTERAFTGEYWDTAEDGIYVDVVS 58
MSRB_ECOLI  -----------MANKPSAEELKKNLSEMQFYVTQNHGTEPPFTGRLLHNKRDGVYHCLIC 49
MSRB_BACSU  -------------MAYNKEEKIKSLNRMQYEVTQNNGTEPPFQNEYWDHKEEGLYVDIVS 47
                               :    *.  *: * :: .**  : ..  .  . * *    .

DR_1378     GEPLFSSLDKYDAGCGWPSFTQPIPDVALTENTDYKIGYARTEVRSASADSHLGHVFPDG 116
Deide_04050 GEPLFSSRDKYDAGCGWPSFTRPIQNMSLTENTDYKIGYPRTEVRSPVADSHLGHVFPDG 113
DGo_CA0919  GEPLFSSKDKYDAGCGWPSFTRPLKDVSLTENTDYKIGYARTEVRSRGVDSHLGHVFPDG 117
Dgeo_2072   GEPLFSSLDKYDAGCGWPSFTRPIPGVTLTERTDHKIGYPRTEVRSGLADSHLGHVFPDG 112
Deima_1441  GEPLFSSTDKYDAGCGWPSFTRPITDHGITERVDRQYGMVRTEVRSTQADSHLGHVFDDG 111
Deipe_4299  GNLLFDSSSKYHSGCGWPSFTEAVAPSAVELLEDRSHGMIRTEVRCARCHSHLGHVFDDG 120
Deipr_1900  GEPLFSSRDKYDAGCGWPSFTRPIAQ--LTEKTDFKLMYPRTEVRSQVADSHLGHVFPDG 116
MSRB_ECOLI  DAPLFHSQTKYDSGCGWPSFYEPVSEESIRYIKDLSHGMQRIEIRCGNCDAHLGHVFPDG 109
MSRB_BACSU  GKPLFTSKDKFDSQCGWPSFTKPIEEE-VEEKLDTSHGMIRTEVRSRTADSHLGHVFNDG 106
            .  ** *  *:.: ****** . :    :    * .    * *:*.   .:****** **

DR_1378     PRDRGGLRYCINSAALRFVPLSELDAQGYGQYRALFEGRQG 157
Deide_04050 PQEHGGLRYCINSASLRFVPVGQLEAEGYADYLPLFR---- 150
DGo_CA0919  PQEEGGLRYCINSASLRFVPAGQLEAEGYGEYAPMFR---- 154
Dgeo_2072   PSEAGGLRYCINSAALRFVPLERLEEEGYGEYRKLFEQQD- 152
Deima_1441  PREHGGLRYCINSAALRFIPVEQLEAEGYGEYQRLFVGG-- 150
Deipe_4299  PRDRGGQRYCMNSVALNLEER-------------------- 141
Deipr_1900  PQDQGGLRYCINSAALRFVPLSQLDAEGYGEYRQLFG---- 153
MSRB_ECOLI  PQP-TGERYCVNSASLRFTDGENGEEING------------ 137
MSRB_BACSU  PGP-NGLRYCINSAALRFVPKHKLKEEGYESYLHLFNK--- 143
            *    * ***:**.:*.:
```

Figure S10. Multiple sequence alignment of *Deinococcus* Msr proteins. **(a)** Alignment of *Deinococcus, E. coli* and *B. subtilis* MsrA sequences. MSRA_ECOLI and MSRA_BACSU, MsrAs from *E. coli* and *B. subtilis*, respectively. **(b)** Alignment of *Deinococcus, E. coli* and *B. subtilis* MsrB sequences. MSRB_ECOLI and MSRB_BACSU, MsrBs from *E. coli* and *B. subtilis*, respectively. Catalytic and resolving cysteines are highlighted in green and cyan, respectively. Extra cysteines present in some sequences are highlighted in yellow. The black lines indicate the consensus sequences proximal to catalytic cysteines and prevalent in MsrA and MsrB.

Figure S11

(a)

```
DR_2242        -----MTLVGQPAPDFTLPA----STGQDITLSSYRGQ-SHVVLVFYPLDFSPVCSMQLP 50
Deide_02430    -----MSLLGQPAPDFTLPS----STGENITLGSYRGQ-KHVVLVFYPLDFSPVCSMQLP 50
Dgeo_0122      -----MSLLGQPAPDFTLPS----TLGEPVTLSSYRGQ-QHVVLVFYPLDFSPVCSMQLP 50
DGo_CA2657     -----MSLVGQPAPDFTLPA----STGQAVTLSSYRGH-SAVVLVFYPLDFSPVCSMQLP 50
Deima_0618     -----MSLLGQSAPDFTLPS----TLGEPITLSSYRGQ-KHVVLVFYPLDFSPVCSMQLP 50
Deipe_1016     -----MSLLGQNAPDFALPS----TEGREIRLSDFKGQ-QHVVLVFYPLDFSPVCSMQLP 50
Deipr_0175     MIASPQALLNQTAPDFTLNAVQPGGLWQPVTLSSYAAAGRWAVLVFYPLDFSPGCTAQVP 60
AHPE_MYCTU     -----MLNVGATAPDFTLRD----QNQQLVTLRGYRGA-KNVLLVFFPLAFTGICQGELD 50
                         :.  ****:*         . : * .: .    .:***:** *:  *  ::

DR_2242        EYSGSQDDFTEAGAVVLGINRDSVYAHRAWAAEYGIEVPLLADMQ--LEVARQYGVAIDE 108
Deide_02430    EYSGRQDDFADAGAVILGINRDSVHAHKAWAADYGIEVPLLADMK--CDVARQYGVTVDE 108
Dgeo_0122      EYSGRQDDFAEAGAVVLGVNRDSVYTHKAWAAEYGIEVPLLADMN--LNVARQYGVAIDE 108
DGo_CA2657     EYSGRQDDFAEAGAAVLGINRDSVHAHKAWAAEYGIDVPLLADMR--LDVARLYGVAIDD 108
Deima_0618     EYSGRQDDFADADTVVLGVNRDSVYTHQAWAAEYGIDVPLLADLN--LAVARAYGVALDE 108
Deipe_1016     EYSGRQEDFAALDTTVLGVNRDSVYTHKAWAAEYGIEIPLLADLN--LKVAREYGVAIDE 108
Deipr_0175     DYSRHAADFDAAGADVLCISRDSVYTHRAWSRELGLQVPLLADMN--LAVAAQYGVALPD 118
AHPE_MYCTU     QLRDHLPEFENDDSAALAISVGPPPTHKIWATQSGFTFPLLSDFWPHGAVSQAYGVFNEQ 110
               :      :*   .:  * :. .   :*: *: : *: .***:*:     *:  ***   :

DR_2242        RGISGRAVFVIDREGVVRYQHVEEQTGQYTVRPGAVLEQLRGL-- 151
Deide_02430    RGVSGRAVFLIDREGVVRFEHVEAKPSEYTIRPEVVLSKITEL-- 151
Dgeo_0122      RGISGRAVFLIDKGGVVRFEYVEAQTGDYTVRPELVLAKLAEL-- 151
DGo_CA2657     RGISGRAVFLIDKEGVIRYAHVEEKTSDYTVRPEQVLAQLRAL-- 151
Deima_0618     RGISGRAVFLIDKGGVVQFEHVEEKTGDYTVRPADVLDRVATLR- 152
Deipe_1016     RAISKRAVFLIDKDGVVRFEGVEPSTGDYSVRPEQVLEQIRTL-- 151
Deipr_0175     QGCARRAIFVVGPDGRVRWLHLEEDPTEVTLSAREVLAQLP---- 159
AHPE_MYCTU     AGIANRGTFVVDRSGIIRFAEMKQPGEVRDQR--LWTDALAALTA 153
                . : *. *::.  * :::  ::                :
```

(b) (continues on next page)

```
DR_1765          ----MPTT--------------QPRLSFLAVPTEDNAHEGVKK-LWSKAEANM--GFVPN 39
Deide_13030      ----------------------MNRISWLEVPDEHSAPEGVRK-LWGKAEANL--GFVPN 35
Dgeo_1446        MTTTQPEA--------------KDRISSLPVPDATQVPEGVRK-LWAKAEANI--GFVPN 43
DGo_CA1027       --MTHNPD--------------AAPISFLPLPTEDTAPEGVRR-LWNKAQANL--GFVPN 41
Deima_0298       ----------------------MPSLSFLPVPTEAEVTPEIAT-LWRKAHGAL--GFTPN 35
Deipe_3296       -------M--------------NPEISRLRLPRREDLPQEARE-LMSAAEDKF--SFVPN 36
Deipe_4199       -------M--------------KQFASWISVIPYEEATGKLKI-LYDRIKGPG--NEIDN 36
Deipe_3903       -------------------------MPRISPINPETASPELQQTLYTVKSKMG--GKLPN 33
Deipe_3878       -------------------------MARIDQVTPEQATGRAKQLLDAV-QNQR--GMTPN 32
Deipe_3900       --MS-KPY--------------HLQ---LPQVTLENAEPEARE-VLERAKRQV--GRLPN 37
Deipr_2741       --MT-VPD--------------SSKARRLSVHTVESAPEGSRAQLEAVQKRNG--GYLPN 41
Deide_1p00700    --MSRVTA--------------PSRLPWMARLA-------SRALTWR-----F--GKPFP 30
AHPD_MYCTU       --MSIEKLKAALPEYAKDIKLNLSSITRSSVLDQ--------EQLWGTLLASAAATRNPQ 50

DR_1765          VF---------------RAQALNGEQFLAWWNY-F-----------------NLLVNKEG 66
Deide_13030      VF---------------RAQALNGEQFLAWWNY-F-----------------NLLVNKEG 62
Dgeo_1446        VF---------------RAQAVNGEQFLAWWNY-F-----------------NLLLNKEG 70
DGo_CA1027       VF---------------RAQALNGEQFLAWWGY-F-----------------NLLLNKEG 68
Deima_0298       VF---------------RAQALNPAQFWAWWKY-Y-----------------DLLMNKEG 62
Deipe_3296       VL---------------RAWAVRPDHLVKWRAY-Y-----------------DLIMQGES 63
Deipe_4199       IM---------------LTHSLRPHSMEGHMAL-Y--------------K--NVLHHHGN 64
Deipe_3903       LV---------------TTFAQSPAALNGYLGF-G-----------------N--GVQSG 58
Deipe_3878       IL---------------QVMALSPNVLDAYLKF-T-----------------G--A-LGQ 56
Deipe_3900       MY---------------LLMANHPGLLETYLNG-Y-----------------D-HLRKSS 63
Deipr_2741       LL---------------GVLSNSPTVLESYLTL-------------------S-KLNGKT 66
Deide_1p00700    TV---------------ALLSHHPAYPVPYLFM-A-----------------GIYGNGST 57
AHPD_MYCTU       VLADIGAEATDHLSAAARHAALGAAAIMGMNNVFYRGRGFLEGRYDDLRPGLRMNIIANP 110
                                                     :
```

```
DR_1765       GLSNAERELLAVVVSGLNRCVYCAVSHGAALREFSG----DAVKADAVAVN----WRQAE 118
Deide_13030   QLSNTDRELLAVVVSGMNRCVYCAVSHGAALREYSA----DPVLADTVAIN----WRHAN 114
Dgeo_1446     YLTNAERELVAVVVSGVNRCLYCAVSHGAALREFLG----DPQKADAVAVN----WRHAD 122
DGo_CA1027    FLTVAERELLAVVVSSLNRCVYCAVSHGAALREATG----DARTADGAAVN----WRQAA 120
Deima_0298    HLPPLEREMVATVVSSLNRCVYCLVSHASAVRVLSG----DVRLADTLAID----YRQAD 114
Deipe_3296    SLTRTQREMIAVAVSSVNRCVYCSTTHPAFLRLELQREGRDPLLAHVLQSNPDHALHDER 123
Deipe_4199    SLPKWLLEVTGVYVSLLNGCEYCVEHHHAGLTRLLRDDDR----AHAIREALENQTPEQV 120
Deipe_3903    TLSPQLREQIALVVAQANGCDYCLAAHSMLGKMRGLDDQNV---RQA-R------QG-QA 107
Deipe_3878    TLSPRLREQIAVLVAQLNNCGYCLAAHTAAAKRAGIDESEL---QAN-Y------QA-DS 105
Deipe_3900    RLNPVEQEVVFLTLSRENSCEYCTSVHSFIADQMSKVPTEV---TDAIR------DGRPI 114
Deipr_2741    SLTPDEREVVQLMAATTHGCSFCVAGHTLTAQKTTKLSAED---IEALR------GHKTL 117
Deide_1p00700 KLNPTTKALVSHLVAQLNGCAFCIDLGQRVARDKGLDTSKLQW-VLAF-------RERPE 109
AHPD_MYCTU    GIPKANFELWSFAVSAINGCSHCLVAHEHTLRTVGVDREAI---FEALK-------AAAI 160
               :            :  : * .*

DR_1765       LSEREQAMCAYAEKLTLRPAEMTEADLAPLRAAGLSDEAILEAVQVIAMFNMTNRVSSAL 178
Deide_13030   LGARERAMCAYAEKLTRTPEAMTQEDLTPLRQAGLSDPQILELVQVVGMFNMTNRISSAL 174
Dgeo_1446     LTEREQALAAYAEKLTRHPAEVTAADLEPLRAVGLDDHQIMELVQVIGMFNLTNRVSSAL 182
DGo_CA1027    LSPREQAMCAYAEKLTLRPAEMTEADLAPLRGEGLSDAQILELVQVVGMFNLTNRVSSAL 180
Deima_0298    LTARQRAILDYAAHLTRHPDRASRDDLTPLRDAGLDDHAILELTQVVGMFNATNRISSAL 174
Deipe_3296    FTPLERALLSFALDLTLRSHQLGQAHVEALRTAGLSDEGIFDAAQTAAMFNFTNRLANAT 183
Deipe_4199    FEGKELAILRYARALTETPASLHRDSLADMRAAGMDDGEILEVNQVVSYFAYANRTVLGL 180
Deipe_3903    SDPKVQAALGFAQAIVEQRGRVTNMDLQAIRDAGYNDEEIVEITVNVAYNILTNYLNNVA 167
Deipe_3878    GDAKTKAALQFARIVTLERGQIREDDLRAVLLAGYSEQEVLEIIAHVALSVFTNYISNTT 165
Deipe_3900    PDARLEALRTFVRVMHDTRGRPDQAAAQAFFDAGYSEQHILDVILAIAVKTISNYANHVF 174
Deipr_2741    QDSKLAALASYTSAVIANRGAVSDEELQAFFDAGYDQAQALEVVLGVGLATICNFGNNVA 177
Deide_1p00700 YSPAERAALEYAWEATQVTAKVSDETYAT-LSSFYSEREIIELTVAVATENFFNRLTGPL 168
AHPD_MYCTU    VSGVAQALATI-EAL--SPS---------------------------------------- 177
                    *

DR_1765       GFVPNPEYHIQSR--------------- 191
Deide_13030   GFAPNAEYHAQARPDSQS---------- 192
Dgeo_1446     GFVPNPEYYRQAR--------------- 195
DGo_CA1027    GFVPNAEYHVMGR--------------- 193
Deima_0298    GFQPNEEYFHLGRTGQ------------ 190
Deipe_3296    GLLPNDEYHAMGR--------------- 196
Deipe_4199    GVTTQGDMLGLSPSDSDDPDNWSHG--- 205
Deipe_3903    DTDIDFPHA---------PPLSSAAD-- 184
Deipe_3878    KPDVEFPPV---------RPLAAAS--- 181
Deipe_3900    NTPLDEAFS---------SREWRGEPVT 193
Deipr_2741    QTTLNPELE---------PYRWDGLS-- 194
Deide_1p00700 NIESQGFCALPDRQ----P--------- 183
AHPD_MYCTU    ----------------------------
```

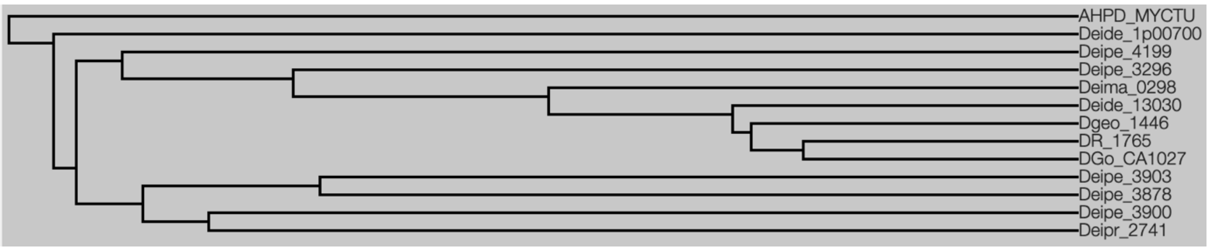

Figure S11. Multiple sequence alignment of *Deinococcus* AhpE and AhpD proteins. **(a)** Alignment of *Deinococcus* and *M. tuberculosis* AhpE sequences. AHPE_MYCTU, AhpE from *M. tuberculosis*. Peroxidatic cysteine (green) and other catalytic triad residues (grey) are highlighted. **(b)** Alignment of *Deinococcus,* and *M. tuberculosis* AhpD sequences. AHPD_MYCTU, AhpD from *M. tuberculosis*. Catalytic and resolving cysteines are highlighted in green and cyan, respectively. The residues highlighted in grey in AhpD sequences are involved in the catalytical mechanism. The guide tree showing sequence relationship between the various AhpD-like proteins is included. Alignments and tree were obtained with UniProt ClustalO.

Figure S12

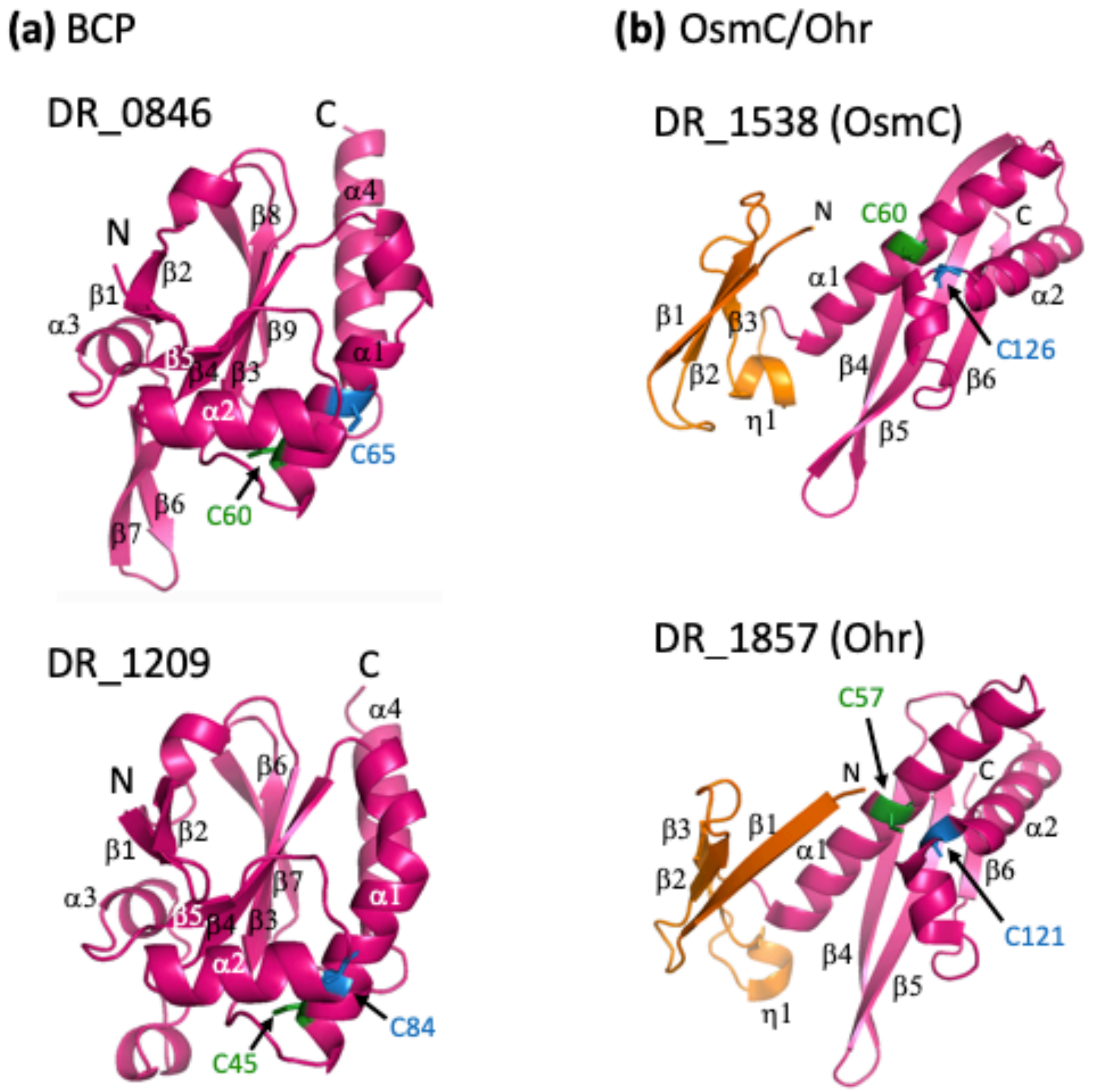

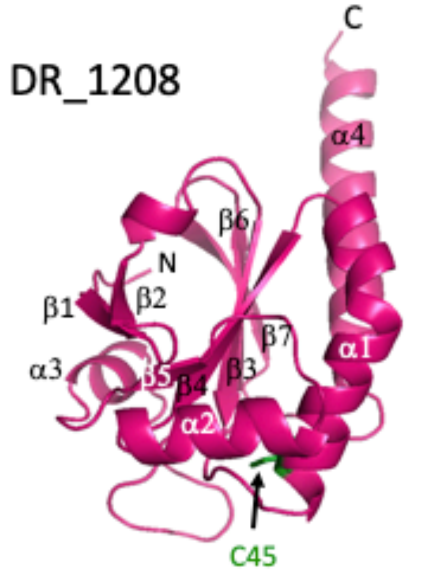

Figure S12. 3D models of BCPs and OsmC of *D. radiodurans.* **(a)** The three BCPs having a Trx-fold. **(b)** Model of OsmC and crystal structure of Ohr (PDB 1USP). Peroxidatic and resolving cysteine residues are shown in green and blue, respectively. The OsmC/Ohr monomer is composed of two distinct subdomains. The N-terminal domain (orange) consists of three β-strands folded into a β-sheet. In the C-terminal domain, helices α1 and α2 (the latter severely kinked) are stacked on a β-sheet formed by strands β4 –β6. The active OsmC and Ohr protein is formed by a homodimer [159]. Models and images were obtained as described in Figure S4.

Figure S13 (panels a to c)

(a)

```
DR_1538      MADIARKASAHWEGDLKSGNGTITTESGVLSQAQYSFKTRFEN--GKGTNPEELLASAHA 58
Deide_16090  MADISRKANAQWFGDLKSGNGTVSTESGALKDNSYSFKARFEQDKAPGTNPEELLAAAHA 60
Dgeo_0526    MADIARKASAQWRGDLRSGQGTIRTESGVLKDAQYSFKTRFEN--GAGTNPEELLAAAHA 58
DGo_CA1241   MADIARKANAQWMGDLKSGKGNISTESGTVKDAQYSFGTRFEN--GVGTNPEELLAAAHA 58
Deima_0667   MANIHRKANAQWTGDLKSGSGTITTPSGVLQAAPYSFRTRFED--QPGTNPEELIAAAHA 58
Deipe_3743   MANIERKANAQWNGDLKSGNGTISSLSGVLSDTPYSFRTRFEN--QPGTNPEELIAAAHA 58
OSMC_ECOLI   -MTIHKKGQAHWEGDIKRGKGTVSTESGVLNQQPYGFNTRFEG--EKGTNPEELIGAAHA 57
               * :*..*:* **:: *.*.: : **.:.   *.* :***     *******:.:***

DR_1538      GCFTMQLSALLAEHGHEIKALDTDATCEMVKDGPGFKINHMHLRVRAQLTGSDQADFEAH 118
Deide_16090  GCFTMQLSAMLAAHGHQPEDLRTEATCEMVKDGPGFKVSTMRLTIRGKVGNIDQAEFEKH 120
Dgeo_0526    GCFTMQLSALLAADGHDPQDLRTEATCEMVREGQGFKVSAMRLVVRGRVGNIDQAEFERH 118
DGo_CA1241   GCFTMQLSALLAAHGHDPQDVRTEATCEMVKDGPGFKVSTMRLQVRGKVGNIDQAEFEKH 118
Deima_0667   GCFTMAFSNVLKTAGYEPRDLATEATLGMSMDG-GPKLTTMHLVVRGKADGLDQTQFQAL 117
Deipe_3743   ACFTMAFSNVLQQAGHAPRTLATDATLGMDASGGGFKIATMHLVVRGSAEGLDQQQFQQL 118
OSMC_ECOLI   ACFSMALSLMLGEAGFTPTSIDTTADVSLDKVDAGFAITKIALKSEVAVPGIDASTFDGI 117
             .**:* :* :*   *.    : * *   :   . *  :  : *  .    . *   *:

DR_1538      VKDAAEKCPLSRIMQGNVEVTHEAILEG 146
Deide_16090  VAQAADACPLSRVMKGNVEITHEAVLE- 147
Dgeo_0526    VQQAAQMCPLSRVMQGNVEITHEAVLE- 145
DGo_CA1241   VAQAADMCPMSRVMKGNVEIVHEAVLE- 145
Deima_0667   AEQAEQGCPVSGALRGNLQITVEATLE- 144
Deipe_3743   AEKAEQGCPVSGALRGNLDITVEAIYEQ 146
OSMC_ECOLI   IQKAKAGCPVSQVLKAEITLDYQLKS-- 143
              .*   **:*  ::.:: :  :
```

(b)

```
DR_1857      -------MANVYTAEATATGGRAGTTRSSDDRLNLDLSVPAEMGG-DGGPGTNPEQLFAA 52
Dgeo_0446    MSGIMSAMSNLYTAEATATGGRTGAVKSSDGRLDLPLSSPRELGG-DGGPGTNPEQLFAA 59
DGo_CA0901   ---MMPLMSNLYTAEAVATGGRAGKTRSTDGRMDLDLSVPAAIGG-DDGPGTNPEQLFAL 56
Deima_2331   -------MSNLYTAEATATGGRAGHARTSDGRLDVDLSVPSEIGG-DGGTGTNPEQLFAA 52
Deipe_0225   ------MSNAIFHTQATAHGGRAGHIETPDHHLDVKLSVPGQLGG-KGGGGTNPEQLFAA 53
Deipr_0815   -------MANLYETTVKTQGARGGTIQSEDGRLQLDLSVPKALGG-DDGQGTNPEQLFAA 52
DGo_CA1828   ----MTQNETLFETTSSAHGGRAGHVEG-EGGLNVRLAVPEAMGG-DGGEGSTPEGLFAA 54
Deipr_0816   -------MQKLYTATATAHGAPGQQVATDDRRIDLPLSLPTELGG-EGGDGTNPEQLLAA 52
Deima_0137   ---MTQELKPLFTATSLVTGGRSGDLQLGRDRTPLTLRPAR-----TRRAGTDPEELFAA 52
OHRA_BACSU   ------MSQPLFTATVSAVGGREGKVISSDRVLELDVAMPGTPRAKKLEKATNPEQLFAA 54
OHRB_BACSU   --------MALFTAKVTARGGRAGHITSDDGVLDFDIVMPNAKKE--GQTGTNPEQLFAA 50
                      :: :   . *.              . :              .: ** *:*

DR_1857      GYAACFQGALGVVSRRQKIDVPADSTITARVGLQK--AGLAFALDVELEGHFPGLSREQA 110
Dgeo_0446    GYAACFQSALGVVARREKVELPEDSTVTARVGLQR--NGLAFALDVELEGHFPGLSSEQA 117
DGo_CA0901   GYAACFQGALAVVARRQKIEIPEGSTVTARVGLER--AGLAFALNVELVGSFPGLEREQA 114
Deima_2331   GYAACFQGALGVAARRQKVSA-DNSEVTAKVGLEK--EGLGFKLNVEIAVRLPGVERDVA 109
Deipe_0225   GYASCFQSAIGVIARQENIEF-GDSTVTALVGLLR--DEQGYGLDVELQITLPGLSREQA 110
Deipr_0815   GYGACFQGAMGLVARRQGIQLPEGSSITATVGMEK--DDVSFLLNAHLVGRFPGMDREQA 110
DGo_CA1828   ALAACFASAMGAVARAENYPEFGDMEVSATAGLSL--DGEAHTLHAALDIKLPGLSREQA 112
Deipr_0816   GYSSCFLAALGIVSKRREVSLSPDIQVQTRLELYE--NGDAYDFKVYLTVSGSDVDAAQL 110
Deima_0137   GYAACYLSALNEVADARRVRVSGAQAL-GQVTLNV-TDAGEYILSVALHVYLPDVAHDEA 110
OHRA_BACSU   GYAACFDSALQLVARTERVKVE--TEVTANVSLLKDEADQGYKLGVTLQVKGEGVSASEL 112
OHRB_BACSU   GYAACFGGALEHVAKEQNIEID--SEIEGQVSLMKDESDGGFKIGVTLVVNTKDLDREKA 108
             . .:*: .*:   :  .          :     :          . : . :      .:

DR_1857      EGLMHAAHEVCPYSAATRNNVDVRLKVRE---- 139
Dgeo_0446    HALMHAAHQVCPYSVALRDNVAVRLKVAG---- 146
DGo_CA0901   QALMDATYNVCPYSVATKGNVETTLTVA----- 142
Deima_2331   EKLVHAAHEVCPYSNATRGNIDVRLSVID---- 138
Deipe_0225   EDLVHKAHQVCPYSRITRGNLDVRLTVVES--- 140
Deipr_0815   QKLMEETLEVCPYSRATKGNMQTSVSVAD---- 139
DGo_CA1828   QHLVDGAKDICAYTRALKGNVDVDYRLHD---- 141
Deipr_0816   QPLLDETLKVCPITRATQGAEIS---VSAG--- 137
Deima_0137   LDLMRAAHAVCPYSHAVRGNIEVTLVAADAPLS 143
OHRA_BACSU   EALVKKAHGVCPYSKATSGNIDVTLEVAE---- 141
OHRB_BACSU   QELVNAAHEFCPYSKATRGNVDVKLELK----- 136
               *:  :  .*  :    .
```

(c)

```
DR_1177     MSTKKTLNVTWLGEQRYLGVSESGHQLLIDNSPV----KVGVSPMEALLGALATCTAYDV 56
Deide_10790 --MKKTLNVTWLGEQRYLGVSESGHQLLIDNSPV----KVGVSPMEALLGALATCTAYDV 54
Dgeo_1268   --MKKTLKVTWLGEQRYVGVSESGHQILIDNSSV----KIGVSPMEALLGALATCTAYDI 54
DGo_CA1763  --MKKTLNVTWLGEQRYLGVSESGHQLLIDNSPV----KVGVSPMEALLGALATCTAYDV 54
Deima_2343  --MKKRLTVHHLGDQRYVGFNETGQQLLIDNSDV----KVGVSPMEALLGAVATCTAYDI 54
Deipe_0234  --MGTKMTMHYLGEQRYVGLNERGQQLLIDASPV----SVGVRPMEALLGALATCSAYDV 54
Deipr_0697  MANTKTVNIDWLGEQRYVGRSENGQQLLIDNSAN----KVGVSPMDALLAALGTCTAYDV 56
Deide_21170 --MQIEVQVRQISPATSQA-TARTHQVMIDRPLEKGGEDRGMMGGEQLLVSLGGCFISNL 57
Deipe_0648  --MQIQVQIHQVGVATAEG-VARTHHVLIDRPTEKGGEDRGMMGGEYLLVALGGCFMSNL 57
YHFA_ECOLI  ----MQARVKWVEGLTFLGESASGHQILMDGNSG----DKAPSPMEMVLMAAGGCSAIDV 52
                    :  :      .     ::::*         . .    : :* : . *   ::

DR_1177     VEIMKKRRTPLASYRIEVEGERADTDPKRYTRITVRHIAAGEGVTAEALSKAAHLSHEKY 116
Deide_10790 VEIMKKRRTPLSTYRIEVEGERADTDPKRYTTITVRHIASGEGITAEALEKAAHLSHEKY 114
Dgeo_1268   VEIMKKRRTPLTAYRIEVEGERADTDPKRYTTITVRHIASGEGLTEEALSKAAHLSHEKY 114
DGo_CA1763  VEVMKKRRTPLSAYRIEVEGERADTDPKRYTHITVRHIASGEGVTEEMLSKAAHLSHEKY 114
Deima_2343  VEIMKKRKTPLSTYRIEIEGDRAEEHPKRYTHIIVRHIAGGAGVTRDALEKAAHLSHEKY 114
Deipe_0234  VGILAKRKTPLSSYRIEVEGERAEEHPRRYTTITVRHIAGGEGVTLEALQKAVQLSHDKY 114
Deipr_0697  VGIMEKRKTPLSSYRIEVEGERADTTPARYTRITVRHIAGGEGVTKEQLEKAAHLSHEKY 116
Deide_21170 LAAIKAREADITDVQLTVTGTL-ESSPSRFSAIEVVVD--AQAQDRALLEKLVEM-SDRA 113
Deipe_0648  LAAIRAREAEIHDVRLEVTGTL-ASAPSRFTEIEVVVG--ARCADPALLEKLVEM-ADRA 113
YHFA_ECOLI  VSILQKGRQDVVDCEVKLTSERREEAPRLFTHINLHFIVTGRDLKDAAVARAVDLSAEKY 112
            :  :   .  :   .: : .      *  :: * :     .       : : ..:  ::

DR_1177     CSVAASLNSEIVVEAELAGEPAAS 140
Deide_10790 CSVAASLNSEIKLETQLE------ 132
Dgeo_1268   CSVAASLNSEIRLETRVE------ 132
DGo_CA1763  CSVAASLNSEISLETRVE------ 132
Deima_2343  CSAAASVNAEITLQVELVETA--- 135
Deipe_0234  CSVVASLNAEIHTDVQLEQAGVPV 138
Deipr_0697  CSVAASLNAEIVLDVRLAEGSEG- 139
Deide_21170 CIVSNTLRPAVALSFRLA------ 131
Deipe_0648  CICTNTLRPAVIPTFRISAIER-- 135
YHFA_ECOLI  CSVALMLEKAVNITHSYEVVAA-- 134
            *     :.  :
```

Figure S13. Multiple sequence alignment of *Deinococcus* OsmC, Ohr and YhfA proteins. **(a)** Alignment of *Deinococcus* and *E. coli* OsmC sequences. OSMC_ECOLI, OsmC from *E. coli*. **(b)** Alignment of *Deinococcus* and *B. subtilis* Ohr. OHRA_BACSU and OHRB_BACSU, OhrA and OhrB proteins, respectively, from *B. subtilis*. **(c)** Alignment of *Deinococcus* and *E. coli* YhfA sequences. YHFA_ECOLI, YhfA from *E. coli*. Peroxidatic and resolving cysteines are highlighted in green and cyan, respectively. The two residues highlighted in grey in Ohr and OsmC sequences are involved in the catalytical mechanism [154].

Figure S14

```
DR_2623     ---------------MSLYDVAIVGAGPVGLAAAIGCKRAGLSYVVLEKGCVVNAIFEYP 45
Deide_23360 --------------MSGLVDVAIIGAGPVGLAAAIACKRAGLSYVVLEKGCVVNAIFEYP 46
Dgeo_2331   --------------MSEMYDVAIVGAGPVGLAAAIACKRAGLSYVVLEKGCVVNAIFEYP 46
DGo_CA0078  --------------MSTLLDVAIVGAGPVGLAAAIACKRAGLSYVVLEKGCVVNAIFEYP 46
Deima_0670  -----------------MFDVAIIGAGPVGLAAAIACKRAGLSYVVLEKGCVVNAIFDYP 43
Deipe_2475  ----------------MMYDLAIVGGGPVGLAAAIAAKRAGLSYTVLEKGCVVNAIFDYP 44
Deipr_1732  MTTEPNPGIPNPEPQGGLYDVAIVGGGPVGLAAAIACKRSGLSYVVLEKGCVVNAIFDYP 60
YPDA_BACSU  ---------------MIQEKAIIIGGGPCGLSAAIHLKQIGIDALVIEKGNVVNSIYNYP 45
BC_1495     ---------------MQKETVIIIGGGPCGLAAAISLQKVGINPLVIEKGNIVNAIYNYP 45
SACOL1520   ---------------MQKVESIIIGGGPCGLSAAIEQKRKGIDTLIIEKGNVVESIYNYP 45
                                  *:*.** **:***  :: *:.  ::*** :*::*::**

DR_2623     TYMGFFTTAPELEIGNHPFVTGHDKPDRRDALMYYRLVTQRENLNVRQYTTVNKVHAAPA 105
Deide_23360 TYMSFFTTAPELEIGNHPMVTGHDKPDRRDALMYYRLVTQREALNVEQYTEVTAVHAAPA 106
Dgeo_2331   TYMTFFTTSPRLEIGNHPFVTQRERPDRKEALHYYRLVAEREALHIEQYTEVTAVHPAPA 106
DGo_CA0078  TYMGFFTTAPELEIGNHPMVTGHDKPDRRDALMYYRLVTQREELKVEQYTEVTKVHAAPA 106
Deima_0670  TYMSFFTTAPELEIGGHPMVTGHDKPDRKDALMYYRLVADREALNIEQYTEVTRVHAAPA 103
Deipe_2475  TYMTFFTTAPELEIGGHPMVSPREKPDRKEALMYYRLVAEREALDLQQYTEVTNVHAAPA 104
Deipr_1732  TDMTFFTTAPELEIGGHPFVSPFDKPKRVDALQYYRKVTQAEGLNVRQYTRVEKVHAAPA 120
YPDA_BACSU  THQTFFSSSEKLEIGDVAFITENRKPVRIQALSYYREVVKRKNIRVNAFEMVRKVTKTQN 105
BC_1495     THQTFFSSSEKLEIGDVAFITENRKPVRNQALAYYREVVRRKSVRVNAFERVEKVQKDGE 105
SACOL1520   THQTFFSSSDKLSIGDVPFIVEESKPRRNQALVYYREVVKHHQLKVNAFEEVLTVKKMNN 105
            *   **::: .*.**.  ::    :* * :** *** *.  . : :. :  *  *

DR_2623     GFTLEIEAQDGTPGVVEARRVVVATGYYDNPLSMGIPGEDSENVSHYYTEAHPFMGLNVT 165
Deide_23360 GFTVQVEKRDGNSGVVEARRVVVATGYYDNPMALGIPGEDSDNVSHYYTEAHPFMGLNVT 166
Dgeo_2331   GFTLAVNRKDGRLGLVEARRVIVATGYFDNPVLLGIPGEDSPNVSHYYTEAHPFWGLKVT 166
DGo_CA0078  GFTLEIERRDGTPGVVEARRVVVATGYYDNPLGLGIPGEDSENVSHYYTEAHPFMGLNVT 166
Deima_0670  GFTVEAERRDGTPGTFEARRVIVATGYYDNPVQLGIPGEDAENVSHYYTEAHPFWKLNVT 163
Deipe_2475  GFTLEVNHKDGTPGVIEARRVVVATGYYDNPVMMGIPGEERENVSHYYTEAHPFWNLKVT 164
Deipr_1732  GFTLQVEAQDGRQDVVEARRVVVATGYYDSPVPLDIPGEDSENVSHYYTEAHPFFGLNVT 180
YPDA_BACSU  N----TFVIETSKETYTTPYCIIATGYYDHPNYMGVPGEDLPKVFHYFKEGHPYFDKDVV 161
BC_1495     AFQVETTKRDGSKEIYIAKYIVVATGYYDNPNYMNVPGEELKKVAHYFKEGHPYFDRDVV 165
SACOL1520   KFT-----ITTTKDVYECRFLTIATGYYGQHNTLEVEGADLPKVFHYFKEAHPYFDQDVV 160
                                  :****:.    : : * :  :* **:.*.**:   .*.

DR_2623     VIGAGNSAADAALDLWRSGVNVTMVVRAPELKSTIKYWVRPDLENRIKEGSIHAHFNSRV 225
Deide_23360 VIGAGNSAADAALDLWRGGANVTMVVRAPELKPTIKYWVRPDLENRIKEGSITAHFNSQV 226
Dgeo_2331   VIGAGSSAADAALDLWRGGAHVTLVVRGAELKPTLKYWIRPDLENRIREGSIQAHFSSRV 226
DGo_CA0078  VIGAGNSAADAALDLWRGGAKVTMVVRAPELKSTIKYWVRPDLENRIKEGSITAHFNSRV 226
Deima_0670  VLGAGNSAADAALDLWRGGANVTMIVRAPELKSTIKYWVRPDLENRIREGSIQAHFNSRV 223
Deipe_2475  VIGAGNSAADAALDLWRGGAQVTMIVRAPSVKNTVKYWVKPDLENRIKEGSIAAHFESRA 224
Deipr_1732  IIGAGNSAADAALDLMNAGANVTMVVRGEGIRSTVKYWVKPNLENRIKEGRIGAHFRSQV 240
YPDA_BACSU  VIGGKNSSVDAALELVKSGARVTVLYRGNEYSPSIKPWILPEFEALVRNGTIRMEFGACV 221
BC_1495     VIGGKNSSVDAALELVKSGARVTVLYRGIEYSPSIKPWILPEFEALVRNGTIQMHFGAHV 225
SACOL1520   IIGGKNSAIDAALELEKAGANVTVLYRGGDYSPSIKPWILPNFTALVNHEKIDMEFNANV 220
            ::*. .*: ****:* ..*..**:: *.     ::* *: *::   :..  *  .* : .

DR_2623     VEIHPEHVVVQGEDGRTFELPTDFTFALTGYRPDLSFLDGLNLATQPDE----CLVLTEN 281
Deide_23360 VEIHPDLVRVQRQDGTTWDLPTHFTFALTGYRPDLSFLSGLGLAQHPDE----CLVLSDH 282
Dgeo_2331   TAILEDRVVVEGPQG-RWELPTDFTFALTGYRPQLSFLADLNLATQADQ----CLALSEH 281
DGo_CA0078  VEIHPEHVRVQREDGTTWELPTDFTFALTGYRPDLSFLAGLDLATQPDE----CLVLDEH 282
Deima_0670  TEIREDSVIVDGPDG-AWELPTHFTFALTGYRPDLSFLEGLGLAQHEDE----CLVLNEH 278
Deipe_2475  VEIGDDYVVVENQSGEKWQLPTDFTFALTGYRPKLDFLAGLGLAQHEDE----CLMLSDT 280
Deipr_1732  VEILPDAVRVQREDGTTFLLPTHFTFAMTGYLPNLDFLEGLDLRTLPDE----CLVLSEH 296
YPDA_BACSU  EKITENEVVFRSGEKELITIKNDFVFAMTGYHPDHQFLEKIGVEIDKE--TGRPFFNEET 279
BC_1495     KEITEHTLTFTVD-GEALTIKNDFVFAMTGYHPDHSFLTKMGVQIDEE--TGRPFYTEDR 282
SACOL1520   TQITEDTVTYEVN-GESKTIHNDYVFAMIGYHPDYEFLKSVGIQINTNEFGTAPMYNKET 279
              *  . :           : ..:.**: ** *. .**  :.:    :      :   :

DR_2623     YESSVPGLFVVGSAGFAGKTNQVFIENGRFHADHAVAEIERQLRSGELRPA------- 332
Deide_23360 YESSVPGLFVAGSAGFAGKTNQVFIENGRFHADVAVAEITRQLGSGVLRQG------- 333
Dgeo_2331   YETSIPGLFVVGSAGFAGRTNQVFIENGREHALLAVAEIERQLGALTEFPALPSPPR- 338
DGo_CA0078  YQSSVPGLFVVGSAGFAGRTNQVFIENGRHHALLAVAEIEQQLGTRDLRPA------- 333
Deima_0670  FESSVPGLFVAGSAGFAGRTNQVFIENGRHHAVVAVEEIVRQLASGTLLAR------- 329
Deipe_2475  FESSVPGLFVVGSAGFAGRTNQVFIENGREHALVALAEIARQLQAVRL---------- 328
Deipr_1732  HESTVPGLFVAGSAGYAGRTNQVFIENGRIHAEEAVAEIARQLAQAEAQATARSGEMA 354
YPDA_BACSU  METNVEGVFIAGVIAAGNNANEIFIENGRFHGGHIAAEIAKRENH------------- 324
BC_1495     METNAENIFIAGVIAAGNNANEIFIENGRFHGDAIAQTIASREK-------------- 326
SACOL1520   YETNIENCYIAGVIAAGNDANTIFIENGKFHGGIIAQSMLAKKQTPLES--------- 328
             ::.  . ::.*  . .. :* :*****: *.      :  :
```

Figure S14. Multiple sequence alignment of *Deinococcus* bacillithiol reductases. Bdr homologs from *Deinococcus* are aligned with Bdr proteins YPDA_BACSU from *B. subtilis*, BC_1495 from *B. cereus*, and SACOL1520 from *S. aureus*. Cysteine residues are highlighted in yellow.

Figure S15

```
DR_1832     ----MTQGQDQVLSPLTTPDEVDQFLKDYPLAAVFK-AGTCHKTMQGFGVLETFLQ-RYE 54
Deide_14700 MTQATQAENQQVLVPLTTPEEVDQFLTEYPLAAVFK-AGTCHKTMQGFGVLETFLQ-RHE 58
Dgeo_1464   MT-QNATQQEQVLVPLTTPDEVDRFLQAYPLAAIFK-AGTCHKTMQGFGVVESFLQ-RHE 57
DGo_CA1021  MT-QTASQDPQVLVPLTTPEEVDQFLQDYPLAAVFK-AGTCHKTMQGFGVLETFLQ-NHE 57
Deima_1446  -------MSEQVLVPLTTPEEVDTFLAEHPLAAVFK-AGTCHKTMQGFGVLETFLK-GHE 51
Deipe_3166  ---------MTQLVPLTTPDEVETFLAQFPLAAIFK-AGTCHKTMQGFGVLETYLA-RHE 49
Deipr_0555  --MTQATQAEQVLLPLTTPEEVQTFLAEYPLAAVFK-AGTCHKTMQGFGVLETFLQ-RHE 56
YtxJ_BACCE  ----------MNMTKLETIEELEVLVEKNEPYVLFKHSTTCPISHGAYTEFQAYCSEERE 50
YTXJ_BACSU  ----------MAKQLIQSEEEFKRIAEQEGVFVFLKHSTTCPISQAAFHEFDAFANQHED 50
SACOL0804   -----------MAIKLSSIDQFEQVIEENKYVFVLKHSETCPISANAYDQFNKFLY-ERD 48
                           : : ::.. .        .:* : **  :  .:  .: :     :

DR_1832     LPVGFIRVVDWRPASNHVAELTGLTHHSPQFILFKDGQAQYEVNNWDITPEALGPVFEQQ 114
Deide_14700 LPVGFIRVVDWRPASNHVAQRTGIVHHSPQFILFRDGEVQFEVNNWDITPEALRPVFDSQ 118
Dgeo_1464   LPVGFIRVVDWRPASNHVAELTGIVHHSPQFILFRNGQPLFEVNNWDITPEALAPVFEAQ 117
DGo_CA1021  LPVGFIRVVDWRPASNHVAQRTGITHQSPQFILFKQGEPQFEVNNWDITPEALSPVFESH 117
Deima_1446  LPIGFIRVVDWRPASNHVAERTGIVHHSPQFILFREGQPVFDVDNWDITPGALTPVFDEF 111
Deipe_3166  LPVGFIRVVDWRPASNHVAERTGVRHHSPQFLLFKNGQSVFDVDNWDITPQALAPVFEAH 109
Deipr_0555  LPVGFIRVVDWRPASNFVAEMTGIQHHSPQLIIFQEGQPRFEVNNWDITPEALAPVFEAL 116
YtxJ_BACCE  VPAYYLYVQDARDVSNRVAEQYSIRHESPQVLYIKDGMVVWNTSHWNIKKDALEENIK-- 108
YTXJ_BACSU  VPAYYLQVQEARPLSNFIAETYGVKHESPQIFIIQNGEVKWHTSHSQITEAAIEQHLS-- 108
SACOL0804   MDGYYLIVQQERDLSDYIAKKTNVKHESPQAFYFVNGEMVWNRDHGDINVSSLAQAEE-- 106
            :   :: * : *  *: :*:  .: *.*** : : :*   :. .: :*.  ::    .

DR_1832     VPQRSGAAQVDAGDSVEPYRQLMQAYLAGQLSDWAFQDQYVTMFRDDASLRSQREFELLS 174
Deide_14700 VPQRTGSAAIATDDNVEPYRRLMRDFVDGRLNEWAFQDQYVTMFRDDASLRSQREFELLS 178
Dgeo_1464   VPRRSTETAVATDDNVEPYRRLMRAYLDGQLSDWAFQDQYVTLFRDDASLRSQREFEALS 177
DGo_CA1021  VPARSGEGAVATEDNAEPYRRLMHAFLDGQLSEWAFQDQYVTMFRDDASLRSQREFELLS 177
Deima_1446  VPARAAEGQVRTQGNVEPYKRLMQDFLDGRLNEWAFQDQYVTMFRDDASLRSQAEFELLS 171
Deipe_3166  VPLRTDT-AEGISTNIEPYKQLMHQFLNDELNEWQFQEAYVNYFRDDANLRSQREFELLS 168
Deipr_0555  VPARAG-GNVETEGNIQPYLDLMDAYLGGQLNDFEFQDRWVPLFRDDASLRSQHEFELLS 175
YtxJ_BACCE  ------------------------------------------------------------
YTXJ_BACSU  ------------------------------------------------------------
SACOL0804   ------------------------------------------------------------


DR_1832     RLFGDPDAYHGGLHQLGAPQDRGDLKARVQSVLDQLG------- 211
Deide_14700 RLFGDPDAYHGGLHQLGAPQDRGDLRARVQALLQEL-------- 214
Dgeo_1464   RLFGDPDAYHGGLHQLGAPQARGDLKARVQALLNELSSRSLG-- 219
DGo_CA1021  RLFGDPDAYHGGLHQLGAPQERGDLKARVQTLLNDLG------- 214
Deima_1446  RLFGDPDAYHGGLHQLGAPAERGDLRGRVQELLAQL-------- 207
Deipe_3166  RLFGDPDAYHGGLHQLGAPQSRGDLKTRVQELLAQL-------- 204
Deipr_0555  RLFGDPDAYHGGLHQLGAPASRGDLRPRVEQLRTELLALRGQSA 219
YtxJ_BACCE  --------------------------------------------
YTXJ_BACSU  --------------------------------------------
SACOL0804   --------------------------------------------
```

Figure S15. Multiple sequence alignment of *Deinococcus* bacilliredoxins. The BrxC-type bacilliredoxins (also called AbxC for atypical BrxC) from *Deinococcus* are aligned with BrxC proteins YtxJ_BACCE from *B. cereus*, YTXJ_BACSU from *B. subtilis*, and SACOL0804 from *S. aureus*. Active site cysteine is highlighted in yellow.
